# Metal and Covalent Organic Frameworks for Membrane Applications

**DOI:** 10.3390/membranes10050107

**Published:** 2020-05-22

**Authors:** Mingyuan Fang, Carmen Montoro, Mona Semsarilar

**Affiliations:** Institut Européen des Membranes—IEM UMR 5635, Univ Montpellier, CNRS, ENSCM, 34095 Montpellier, France; mingyuan.fang@enscm.fr

**Keywords:** MOF membranes, COF membranes, membrane characterisation, gas separation, liquid separations, fuel cells

## Abstract

Better and more efficient membranes are needed to face imminent and future scientific, technological and societal challenges. New materials endowed with enhanced properties are required for the preparation of such membranes. Metal and Covalent Organic Frameworks (MOFs and COFs) are a new class of crystalline porous materials with large surface area, tuneable pore size, structure, and functionality, making them a perfect candidate for membrane applications. In recent years an enormous number of articles have been published on the use of MOFs and COFs in preparation of membranes for various applications. This review gathers the work reported on the synthesis and preparation of membranes containing MOFs and COFs in the last 10 years. Here we give an overview on membranes and their use in separation technology, discussing the essential factors in their synthesis as well as their limitations. A full detailed summary of the preparation and characterization methods used for MOF and COF membranes is given. Finally, applications of these membranes in gas and liquid separation as well as fuel cells are discussed. This review is aimed at both experts in the field and newcomers, including students at both undergraduate and postgraduate levels, who would like to learn about preparation of membranes from crystalline porous materials.

## 1. Introduction

Membranes could be found in nearly all areas of science and technology since their development is destined to solve problems related to aging population, emerging developing countries, pollution, environmental remediation, and more efficient industrial production to name a few. The wide use of this type of materials in industry is due to the fact that membrane separation processes are more energy-efficient and it does not involve any phase transformation.

According to the International Union of Pure and Applied Chemistry (IUPAC) a membrane is “a structure that has lateral dimensions much greater than its thickness, through which transfer may occur under a variety of driving forces” [1] This definition may sound similar to the concept of thin-film but in this case, IUPAC defines thin-film as “a film whose thickness is of the order of a characteristic scale or smaller” [2]. The difference between a membrane and a thin-film is that a membrane is a thin, film-like structure (not necessarily a solid) that separates fluids (liquids, gases, or vapours), acting as a selective barrier, allowing specific substances to pass through, while retaining others. In contrast, due to the mechanical properties of the thin films, they must be placed on a substrate and does not necessarily fulfil the same function; instead it can be part of a membrane as in the thin-film composite (TFC) membranes.

Membranes can be classified according to different criteria: nature, morphology, bulk structure, geometry configuration as well as the size of the separated particles [3,4]. Commonly the membranes are grouped into biological or synthetic membranes. While biological membranes are within or around a cell in a living organism, recognising what is necessary for the cell to receive or block for its survival, synthetic membranes are at the heart of a wide range of key industries such as food, biotechnology, electronics, and energy. They can be produced from organic material such as polymers (polyvinylidene fluoride (PVDF), polyamide, polyimide, etc.) or from inorganic materials like metal oxides and ceramics. An ideal membrane should present the following industrial requirements: (1) be as thin as possible with good mechanical properties to lower energy consumption and cost; (2) show high pore density to maximize the membrane porosity and thus the flux and permeability; (3) be selective due to a controlled morphology at the nanometre scale; (4) use a universal preparation strategy providing a broad range of pore sizes with a sharp control especially between 1 and 100 nm; (5) good chemical and thermal stability and; (6) have tuneable dynamics to afford in-situ control over membrane parameters (pore size, flux, etc.) [5].

Currently, most of the commercial membranes are based on polymeric materials due to their high processability into viable membrane structures and the diverse polymers available, as well as the capability to synthesising novel polymer structures. Nevertheless, polymers present some limitations related to their low thermal and chemical stabilities, low selectivity, and short lifetimes. In this sense, inorganic membranes show higher stabilities and can feature perfectly ordered pores but the range of pore size accessible is restricted and exhibits rather small surface areas. So, given the huge quantity of membrane applications, it is of high interest to explore new nanostructured materials with specific properties and morphologies to offer powerful tools for the preparation of membranes with improved features to existing ones. Membrane-based separation used to be dominated by the porosity of the material so it is a key factor to take into account during the search for new alternative materials [6]. Therefore, attention of the scientific community must be in porous materials that are solids containing empty voids, which can host other molecules.

**Figure 1:**
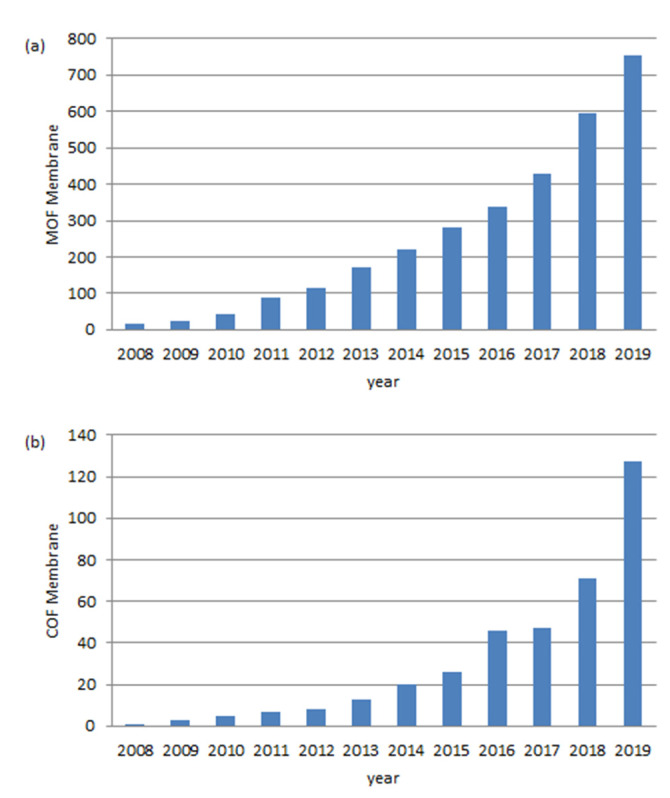
Number of publications per year (**a**) “Metal Organic Framework membranes” and (**b**) “Covalent Organic Framework membranes”. Data obtained from the Web of Science.

Over the past decade, interest in the field of porous materials has grown tremendously because of their outstanding performance and broad applications in gas storage and separation, heterogeneous catalysis, energy, optoelectronics, sensing, and drug delivery. Chemists have found ways to prepare a wide variety of porous materials; however, the synthesis of porous frameworks with discrete pores has proven to be difficult until the inception of the concept of reticular chemistry, which uses topologically, designed organic, and inorganic building blocks linked by strong bonds to make crystalline open frameworks [7,8,9,10]. Metal-Organic Frameworks (MOFs) [11,12] and Covalent-Organic Frameworks (COFs) [13] are two kinds of crystalline porous materials obtained applying this chemistry. MOFs materials are constructed from a metal ion or a cluster of metal ions and an organic linker or bridging ligands through strong coordination bonds [14]. To date, more than 60,000 MOFs with different compositions, crystal structures, and morphologies have been reported due to the possibility of wide variation of metal ions and organic linker combinations [15,16]. On the other hand, COFs are inspired from MOFs and synthesised via reversible strong covalent bonds between light elements such as boron, carbon, nitrogen, and oxygen [7,17,18]. Pure organic components of COFs give them lower density and better compatibility with other organic materials. But both crystalline porous materials are characterised by permanent porosities with very high surface areas, high thermal stabilities, and exceptional chemical stabilities in organic and aqueous media, acids, and bases. Functionality and utility of these structures are often enhanced over those obtained from polymeric materials; due to their much better-defined pore structures with narrower size distribution, increased pore stability, and the tuneable pore sizes depending on the dimensions of the building blocks used. These properties make them both excellent candidates for many applications in fields of gas storage [19], catalysis [20,21], electrochemistry [22], gas separation [23], sensors [24,25,26], and medicine [27], among others. Most of these applications require that the material be previously processed. In this regard, very recently Zamora and coworkers have discussed the strategies used for COF processability [28]. As it can be seen in Figure 1, in the last decade there has been an almost exponential growth in the number of papers published on MOF and COF membranes. This is due to the enormous progress made in the use of MOF and COF in the separation processes using membranes. To date several reviews on MOF membranes have been published focusing on their general and specific applications [29,30,31,32,33,34]. In the case of COF, there is only one review by Van der Bruggen based specifically on COF membranes for separation applications [35]. Very recently, Xu and coworkers have published a review on the preparation of ultrathin MOF and COF membranes for separation applications [36]. Apart from this review, there are no comprehensive reports gathering the work performed on both COF and MOF membranes for membrane applications. In this regard, we collect the work reported on MOF and COF membranes in this manuscript, so that it serves as a base for researchers new to the subject as well as a quick update for the experts in the field.

This review is divided into three parts. In the first part, the most conventional methods used in the preparation of MOF and COF membranes including free-standing membranes, thin-film composite (TFC) membranes, MOF/COF composites membranes, and mixed matrix membranes (MMMs) are gathered. In the second part the main characterisation techniques used to determine membrane properties are discussed. Moreover, unexplored and potential characterisation techniques that could be used for such membranes are introduced. Finally, the most recent and relevant reports on MOF and COF membranes for gas separation (CO_2_ recovery, H_2_ purification and recovery, and hydrocarbon separation), liquid separation (water treatment, organic solvent nanofiltration, and separation of small molecules in liquid mixtures), and in fuel cells are described. From our point of view, this review would serve as a good starting point to learn about the use of MOF and COF material in membrane science.

## 2. Preparation Methods

The fabrication of membranes free of pinhole defects, grain edge defects, inter-crystalline, and intra-crystalline cracks are a major challenge. This section reviews the methods used for the preparation of MOF and COF membranes including thin-film composite membranes, MOF-COF composite membranes and mixed matrix membranes based on MOFs and COFs.

### 2.1. MOF Membranes

MOF membrane preparation is quite similar to that used for zeolites. Indeed, the majority of MOF membranes reported since the first report in 2009 [37], are based on zeolite imidazolate frameworks (ZIFs) because of their zeolite-like structure and properties such as high thermal and chemical stability, permanent porosity, and tuneable pore sizes [23,38]. However, due to their poor mechanical stability, there are not many examples of free-standing MOF membranes and most of them are prepared on inorganic or polymeric porous supports with different shapes depending on the final application. Here, we collect the main methods used for MOF membrane preparation accompanied by some recent examples. For readers interested in large scale MOF membrane preparation there is an interesting recent review published by Li [29].

#### 2.1.1. In Situ Growth

**Figure 2:**
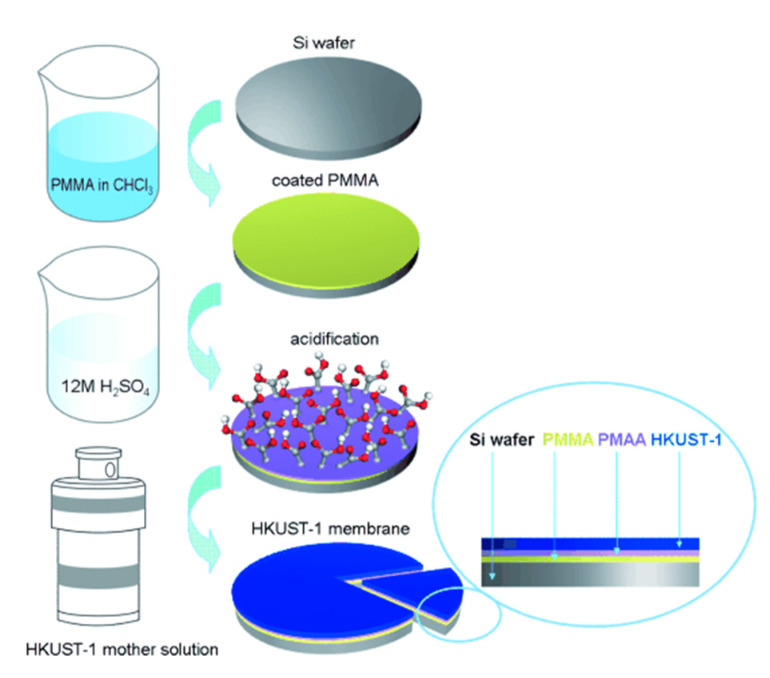
Schematic illustration of the preparation procedure for the free-standing HKUST-1 membrane. Reprinted with permission from [45], Copyright (2012) John Wiley and Sons publications.

In situ or direct growth method is a well-known strategy for the preparation of thin-film MOF membranes. It consists of the immersion of a porous substrate, usually Al_2_O_3_ or TiO_2_, in the precursors solution in which nucleation and heterogeneous crystal growth occurs after the solvothermal, hydrothermal, or microwave treatment applied [39,40]. In order to increase the adhesion of the MOF layers to the substrate, some approaches related to the support surface modification have been tested. For example, Caro and coworkers succeeded in the preparation of thin-film MOF membranes through the surface functionalisation with 3-aminopropyltriethoxysilane (APTES) [41,42] and polydopamine (PDA) [43,44], which acted as covalent linkers between the MOF and the porous substrate. Ben et al. reported a method for the preparation of free-standing MOF membranes with tuneable thickness, from the chemical modification of the surface substrate with poly(methyl methacrylate) (PMMA), which is converted in poly(methacrylic acid) (PMAA) for facilitating the MOF nucleation. Then, the solution of the PMMA-PMAA-coated surface results in the release of the MOF membrane (Figure 2) tested in gas separation [45]. The support surface can also be modified with the same organic linker used for the MOF synthesis as was already demonstrated by McCarthy et al. who grafted imidazolate ligands to α-Al_2_O_3_ supports before growing the ZIF crystals [46].

**Figure 3:**
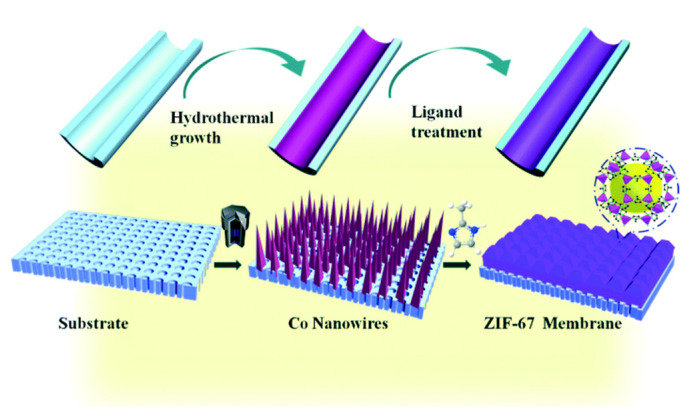
Schematic illustration of the preparation of a pure ZIF-67 membrane by self-conversion of carbonate hydroxide nanowire arrays (Co-NWAs). Reprinted with permission from [53], The Royal Society of Chemistry, Copyright (2018).

Other in situ growth approaches involve the use of a metal surface both as a substrate to support the membrane as well as to act as an ions source for the MOF synthesis [47,48,49,50]. Using the same principle, a metal oxide layer like ZnO can be deposited on a porous support in order to provide active sites prior to the Zn-based MOF membrane formation [51,52]. Recently, Zhang and coworkers showed the preparation of a Co-based ZIF membrane (1.7 μm thickness) on a porous tubular support after the direct transformation of carbonate hydroxide nanowire arrays (Co-NWAs) in a 2-methylimidazole (Hmim) aqueous solution (Figure 3) for H_2_ separation [53].

#### 2.1.2. Seeded Assisted or Secondary Growth

This method allows a better control of the membrane microstructure in terms of nucleation and crystal growth, giving rise to the formation of compact, continuous and thin polycrystalline membranes. Basically, it consists of two steps: (i) seeding support with crystal seeds of the MOF and (ii) growing the crystals up. The first step is crucial for preparing well-intergrown polycrystalline membranes due to a homogeneous nucleation. So, it is very important to have control over the size, amount, location, and orientation of the crystal seeds since it will have influence on the membrane performance [54,55,56,57]. Sun et al. demonstrated the impact of the crystal orientation on gas separation by the preparation of the highly c-oriented NH_2_-MIL-125 membrane with a thickness of 200 nm [57]. Some of the most common techniques used to carry out the seeding method are rubbing [58], dip and spin-coating [54], electrospinning [59], electrospray [60], and layer-by-layer [61].

**Figure 4:**
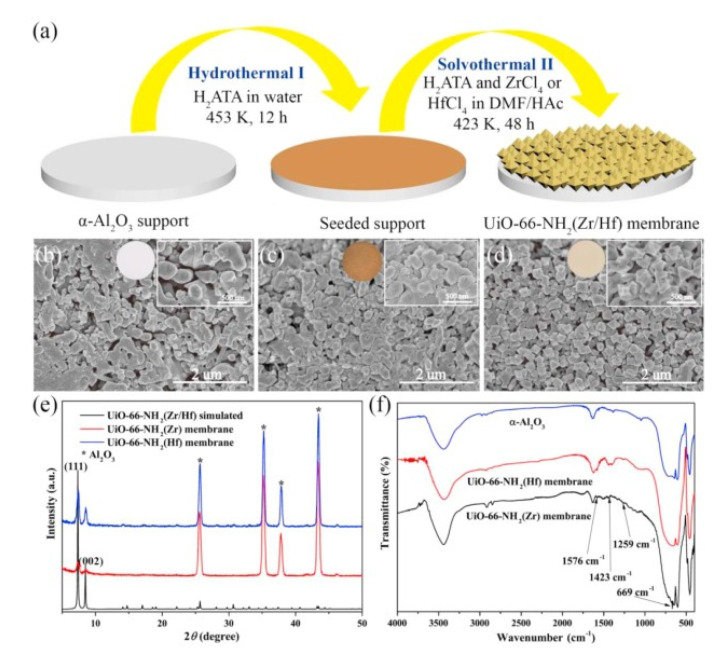
(**a**) Schematic diagram of UiO-66-NH_2_(Zr/Hf) membrane preparation procedure. SEM images of the (**b**) α-Al_2_O_3_ support, (**c**) seed layer, and (**d**) UiO-66-NH_2_(Zr) membrane surface. (**e**) XRD patterns of UiO-66-NH_2_(Zr/Hf) membrane. (**f**) FTIR spectra of UiO-66-NH_2_(Zr/Hf) membrane. Reprinted with permission from [63], Copyright (2019) Elsevier.

Alternately, Lee and coworkers developed a reactive seeding (RS) method for the preparation of continuous MOF membranes on porous alumina supports. As in the in situ growth, in the first step of this approach, the support acts as the inorganic source reacting with the organic precursor to grow the seed layer, which will help the formation of a superior MOF membrane in the secondary growth [62]. This method was further used by Du et al. for fabrication of a uniform, thin, and well intergrown UiO-66-NH_2_(Zr/Hf) MOF membrane (8 μm of thickness) with application in wastewater treatment. In this case, the process started with a uniform seeding layer growth on the support from the reaction between H_2_ATA and α-Al_2_O_3_ under hydrothermal conditions. At a later step, the UiO-66-NH_2_(Zr) membrane or UiO-66-NH_2_(Hf) membrane were prepared from the reaction between 2-aminoterephthalic acid (H_2_ATA) and ZrCl_4_ or HfCl_4_ in the mixed solution of *N’N*-dimethylformamide and acetic acid under solvothermal conditions. After this second step, the yellow colour indicated the formation of the membrane active layer (Figure 4) [63].

#### 2.1.3. Layer-by-Layer Assembly

Layer-by-layer, also known as the step-by-step approach was thoroughly studied by Shekhah et al. for thin-film MOF formation [64]. This method is based on the sequential deposition of organic ligands and metal-oxo coupling units. After each step, the unreacted components were removed by solvent rinsing. One of the most important advantages of the layer-by-layer approach is the possibility to control the thickness of the growing film. Later, the same group applied this method for preparation of a ultrathin (0.5–1 µm) defect-free ZIF-8 membrane on alumina support and used it for separation of mixed gases [65]. Other authors also grew ZIF-8 on different supports applying the layer-by-layer concept but with slight modifications in the preparation method depending on the final application. For example, Kargari and coworkers placed the poly(phenyl sulfone) (PPSU) support at the bottom of a vessel and then soaked alternately with the salt and the organic solutions, the thickness of the ZIF-8 layer can reach about 10 µm per cycle of growth [66]. Very recently, Yang et al. obtained a high-quality ZIF-8 membrane ideal for gas separation, using the layer-by-layer deposition approach with the solvent-free crystallisation method followed by the in-situ heat treatment [67]. Tham et al. adapted this method for the preparation of a Zn benzene-1,4-dicarboxylic acid (Zn(BDC)) MOF on a chemically modified polyacrylonitrile (PAN) membrane using water as the sole solvent. The obtained membrane exhibited good performance in organic solvent nanofiltration (OSN) [68].

#### 2.1.4. Contra-Diffusion or Interfacial Method

**Figure 5:**
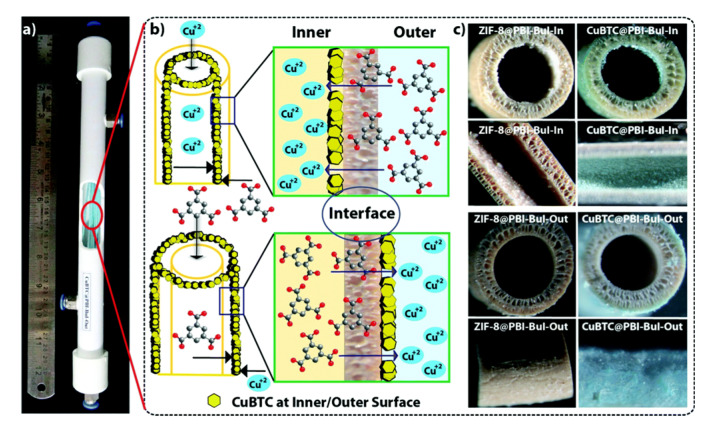
(**a**) Representative digital photograph of a gas separation module (CuBTC growth on the outer surface of the PBI-BuI hollow fibres (CuBTC@PBI-BuI-Out) is seen through the cut); (**b**) schematic for the interfacial synthesis approach of CuBTC@PBI-BuI-In and -Out; and (**c**) microscopic images of the ZIF-8@PBI-BuI-In, ZIF-8@PBI-BuI-Out, CuBTC@PBI-BuI-In, and CuBTC@PBI-BuI-Out composites synthesised. Reprinted with permission from [71], The Royal Society of Chemistry, Copyright (2015).

In 2011 Yao et al. developed this new approach to prepare ZIF-8 films with thickness’ up to 16 µm on a nylon substrate [69]. The method consists of placing the porous substrate between both the metal ion and the organic ligand solution. So, after the slow diffusion of both reagents through the substrate, crystallisation takes place on the membrane surface. Huang et al. used the contra-diffusion method to synthesise a ZIF-71 membrane (2.5 µm of thickness) on an inorganic hollow fibre substrate, ideal for the separation of ethanol–water mixtures [70]. In a similar approach, Biswal et al. showed a scalable method for growing MOFs (ZIF-8 and CuBTC) on either the outer or inner surface of a polybenzimidazole based hollow fibre (PBI-BuI-HF) membrane ideal for gas separation (Figure 5) [71].

#### 2.1.5. Vapour Deposition

**Figure 6:**
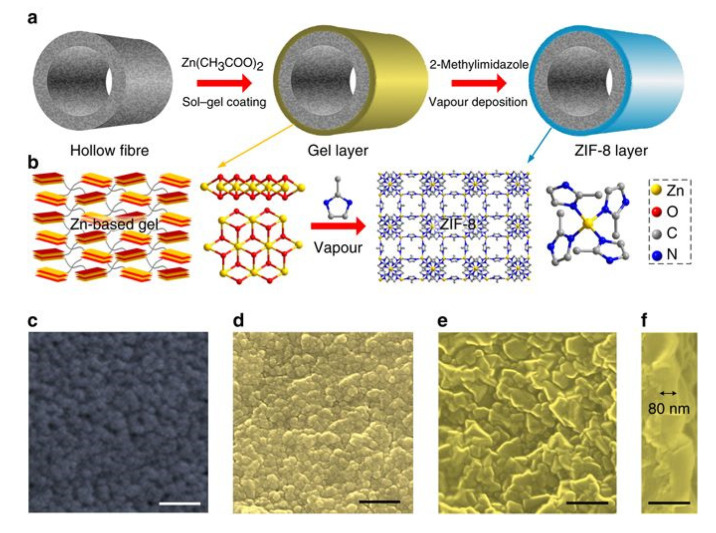
GVD fabrication of an ultrathin ZIF-8 membrane. (**a**) Schematic of the MOF membrane formation process. (**b**) Schematic illustration and chemical structure of Zn-based gel and crystalline structure of ZIF-8. Zn, O, C, and N atoms are depicted in yellow, red, grey, and blue, respectively. H atoms are not presented for clarity. Top view SEM images of (**c**) the PVDF hollow fibre and (**d**) the Zn-based gel layer. SEM images of (**e**) top and (**f**) cross-sectional view of the ZIF-8 membrane prepared with sol concentration of 1 U and coating time of 2 s. The images are coloured for clarity. Scale bar, 200 nm. Reprinted with permission from [72], Copyright (2017) Springer Nature.

Vapour deposition techniques such as gel vapour deposition (GVD) and ligand-induced permselectivation (LIPS) have been used for the preparation of ultrathin MOF membranes. These innovative methods usually are characterised by being simple, reproducible, and scalable. GVD is based on the combination of a modification-free sol–gel coating and solvent-free vapour deposition [72]. Using this approach, Zeng and coworkers got to prepare a MOF membrane module (30 polymeric hollow fibres with a membrane area of 340 cm^2^) without deterioration in selectivity (Figure 6). Li et al. prepared a nanometre-thick ZIF-8 membrane through GVD with excellent gas separation performance [73].

Tsapatsis and coworkers used LIPS to transform an impermeable layer of ZnO obtained by atomic layer deposition (ALD) and deposited on an alumina support in a ZIF-8 membrane that was tested for hydrocarbon separation [74]. In a similar approach, Zhang and coworkers prepared a 2D Co-ZIF nanosheet membrane on a porous tubular substrate via LIPS with major improvement in H_2_/CO_2_ separation [75].

### 2.2. COF Membranes

The following part focuses on COF membrane preparation methods. Due to the organic COF nature, the methods explored are very similar to the one used for polymeric membrane preparation.

#### 2.2.1. In Situ Growth

**Figure 7:**
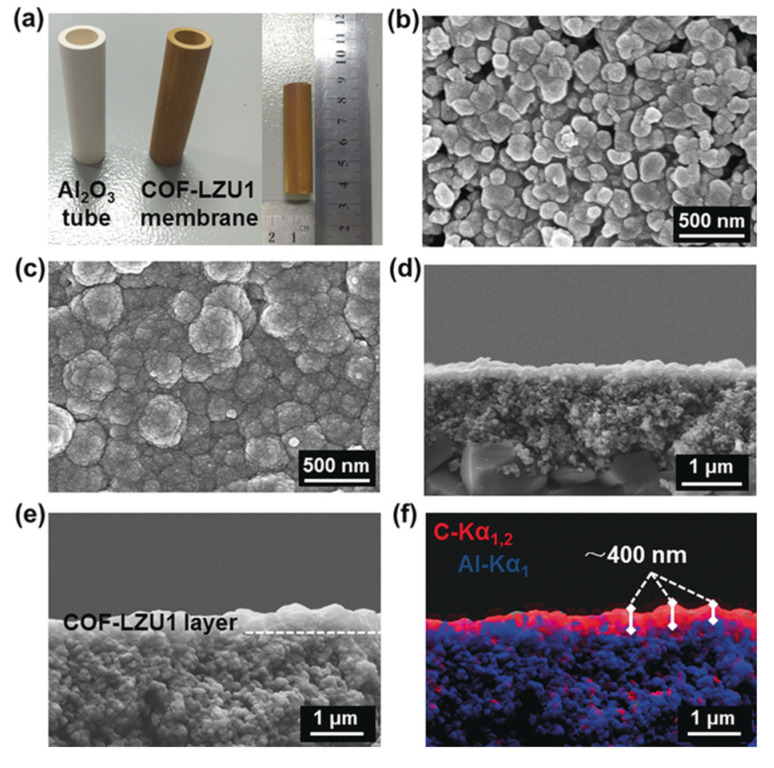
(**a**) Photographs of an untreated Al_2_O_3_ tube and a tubular COF-LZU1 membrane. (**b**,**c**) Top-view SEM images of an untreated Al_2_O_3_ tube (**b**) and a tubular COF-LZU1 membrane (**c**). (**d**,**e**) Cross- sectional SEM images of an untreated Al_2_O_3_ tube (**d**) and a tubular COF-LZU1 membrane (**e**). (**f**) EDXS (Energy-dispersive X-ray spectroscopy) mapping of the cross-section of a tubular COF-LZU1 membrane and corresponding elemental distributions. C is the tracer for the COF layer, and Al is the tracer for the ceramic tube. Reprinted with permission from [78], Copyright (2018) John Wiley and Sons publications.

Using the in situ growth method, Gao and coworkers prepared for the first time a 3D COF membrane on a porous α-Al_2_O_3_, improving their previous results on COF-5 membrane preparation [76,77]. Specifically, the surface functionalisation of the α-Al_2_O_3_ facilitated the adhesion of the COF to the support by the imine condensation reaction between the amino groups of APTES and the aldehyde groups of the monomer during the solvothermal reaction. The resulting compact, uniform, and well-intergrown 3D COF-320 membrane had a thickness of about 4 µm. Caro and coworkers prepared a continuous and defect-free 2D imine-linked COF-LZU1 membrane (thickness of only 400 nm) on the outer modified surface of alumina tubes using in situ solvothermal synthesis. The robust membrane obtained, showed excellent water permeability as well as reasonable rejection rates (above 90%) for dye molecules (Figure 7) [78].

#### 2.2.2. Solution Casting

**Figure 8:**
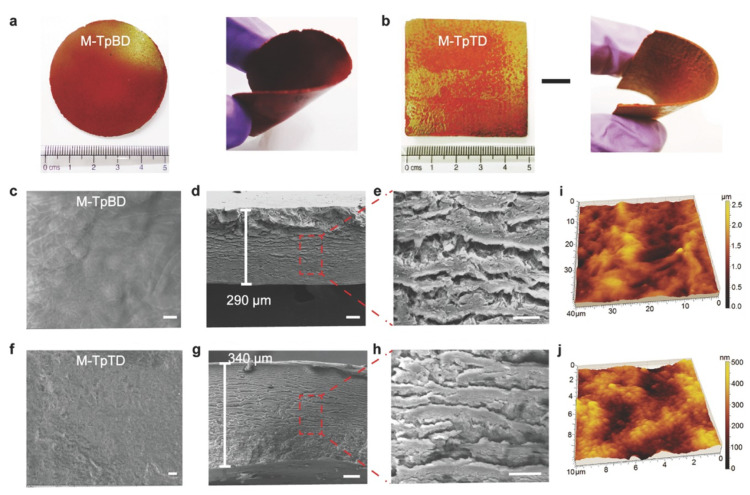
(**a**,**b**) Photographs of M-TpBD and M-TpTD demonstrating the membrane flexibility. (**c**,**f**) SEM images showing surface of M-TpBD and M-TpTD COMs, which also infer that the membrane surface is free from defects and cracks; (**d**,**g**) is the cross-section; (**e**,**h**) is the corresponding zoomed view of M-TpBD and M-TpTD respectively; (**i**,**j**) AFM images of M-TpBD and M-TpTD respectively showing the surface roughness recorded on the top of a silicon wafer (scale bars: (**c**,**f**) 5 μm; (**d**,**g**) 50 μm, and (**e**,**h**) zoomed view 10 μm). Reprinted with permission from [79], Copyright (2016) John Wiley and Sons publications.

Banerjee et al. developed a new methodology for the fabrication of free-standing, flexible, and highly porous COF membranes based on ketoenamine COFs (Figure 8). This cost effective and highly scalable procedure consists of baking the reagent dough that previously had been knife-casted onto a glass plate. The obtained defect- and crack-free COF membrane, with a thickness ranging from 200 to 700 µm, was tested for selective molecular sieving [79]. Using a similar approach, the same group prepared three different COF membranes with a thickness > 100 µm by the slow baking of symmetrical organic linkers in the presence of amino *p*-toluene sulfonic acid (PTSA·H_2_O) and water at moderate temperature for three to four days. In this case, the co-reagent PTSA·H_2_O, besides improving both the porosity and the crystallinity of the membranes, it acted as a proton transporter resulting in high values of proton conductivity [80].

#### 2.2.3. Layer-by-Layer Assembly

**Figure 9:**
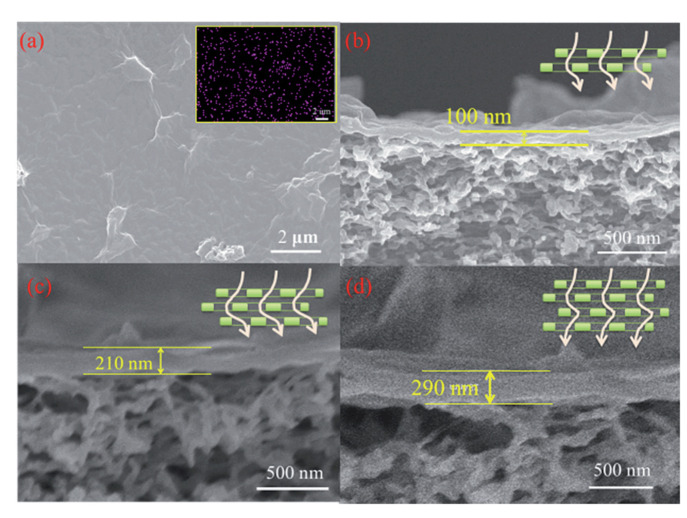
Top view SEM image of (**a**) the 100 nm membrane, and cross-sectional SEM images of the membranes with different thickness 100 nm (**b**), 210 nm (**c**), and 290 nm (**d**). The inset in (**a**) is the corresponding nitrogen EDS mapping, and the inserts in (**b**–**d**) are corresponding schematic representations of different numbers of restacked layers. Reprinted with permission from [82], The Royal Society of Chemistry, Copyright (2016).

Layer-by-layer assembly is also a promising bottom-up strategy for the preparation of ultrathin COF membranes. This approach is based on the deposition of 2D nanomaterials with nanosheet morphology, which can act as building blocks for membrane construction. For example, Li et al. used a solution of COF-1 nanosheets for coating a macroporous α-Al_2_O_3_ substrate with a thin SiO_2_–ZrO_2_ intermediate layer to obtain a membrane free of crack and pinholes with an extremely permeable performance [81]. Yin et al. were pioneered in the preparation of ultrathin (<300 nm) COF membranes from a mixture of covalent triazine-based framework-1 (CTF-1) and graphene oxide (GO) nanosheets in water, which was restacked by filtration. The interactions between the functional groups in GO and the COF sheets led to the formation of continuous and dense ultrathin membranes (Figure 9) [82]. A similar approach was reported by Tang et al. for preparation of a defect free TpPa/GO membrane for H_2_/CO_2_ separation [83].

The deposition of the layer on the support can also be carried out by spin-coating. Pan and coworkers fabricated a dual-layer by the spin-coating of a 2D COF layer on a porous hydrolysed polyacrylonitrile (HPAN) substrate, which was spin coated with bio-inspired calcium alginate (Alg-Ca) layer. The double functionality of this membrane allowed, on one hand, to increase the adsorption of water molecules and, on the other, to serve as a molecular sieve in the alcohol dehydration process due to the structure of the COF [84].

#### 2.2.4. Interfacial Polymerisation (IP)

This technique is very useful for the preparation of polymer thin films like polyamides and polyesters in the bulk scale [85,86]. It consists of the reaction of the monomers at the liquid–liquid interface. So, the diffusion of each of the monomers from the immiscible phase (usually aqueous and organic) will lead to the formation of free-standing thin-films. This layer can be deposited onto a porous substrate giving rise to a thin-film composite (TFC). The main benefits of this technique are related to its scalability and its capacity to produce thin separating active layers (<250 nm) with relatively high water permeability [87]. However, the amorphous nature of polymers makes the obtained TFC lack ordered, tuneable pore structures, which could be resolved with the use of crystalline materials. Pioneer work by Dey et al. used the IP strategy to prepare different TFCs based on free-standing imine-based COF films with permanent microporosity. For this, they decreased the diffusion rate at the interface by using amine-*p*-toluene sulfonic acid (PTSA) salt instead of free amine [88]. Mariñas and coworkers used IP for the preparation of a polyimine COF TFC nanofiltration membrane but in this case, both monomers were dissolved into the organic phase whereas the aqueous solution contained the catalyst [89]. Dichtel et al. used also IP to prepare TAPB-PDA COF free-standing membrane with large-area (several cm^2^) and tuneable thickness (2.5 nm to 100 mm) via changing the initial monomer concentration. This COF films could be easily transferred onto polyethersulfone supports and showed enhanced rejection of Rhodamine WT, a model water contaminant [90].

Alternatively, Wang and coworkers developed a new strategy based on IP that allows the fabrication of the COF selective layer directly on a support. This approach avoids the tedious transference of the thin-film layer onto the support surface as well as the long reaction times [91].

#### 2.2.5. Langmuir−Blodgett (LB) Method

Langmuir−Blodgett (LB) is a potential method for preparing membrane with controllable thickness and large dimensions that can be easily transferred to different support surfaces. Lai et al. were pioneers in the preparation of a crystalline TFP-DHF 2D COF membrane using this method. They spread a solution of the two monomers in toluene on the surface of water. After complete evaporation of the solvent, the surface layer was compressed and trifluoroacetic acid was added dropwise. Finally, a continuous and yellow TFP-DHF-COF thin-film was formed on the water−air interface. These thin film layers were transformed to different supports for membrane application [92].

### 2.3. COF-MOF Composite Membranes

**Figure 10:**
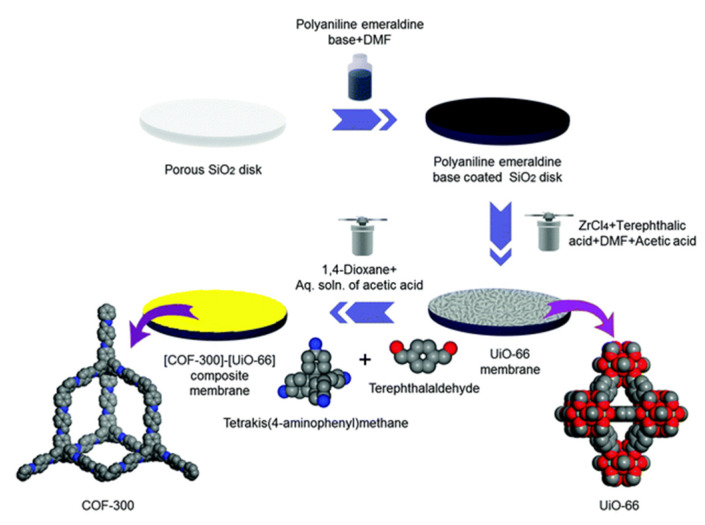
Strategy for the fabrication of the [COF-300]-[UiO-66] composite membrane. Zirconium, carbon, nitrogen, and oxygen atoms are shown as cyan, grey, blue, and red, respectively. Hydrogen atoms are omitted for clarity. Reprinted with permission from [94], The Royal Society of Chemistry, Copyright (2018).

Considering the molecular sieving properties of MOFs and COFs, Fu et al. fabricated novel COF-MOF composite membranes with high performance in gas separation. Specifically, these membranes were prepared by the application of two consecutive in-situ growth methods. One for the synthesis of COF-300 on a modified polyaniline porous SiO_2_ and the second one for growing ZIF-8 or Zn_2_(bdc)_2_(dabco) MOFs using the COF-300 membrane as support. MOF and COF layers were adhered due to both zinc cation with the amine group and hydrogen interactions [93]. Following a similar approach, the same group prepared a [COF-300]-[UiO-66] composite membrane, which showed good performance in H_2_/CO_2_ separation (Figure 10) [94].

### 2.4. Mixed Matrix Membranes (MMMs) Based on MOFs and COFs

Another typical strategy for the preparation of MOF and COF membranes consists of the use of these materials as porous fillers in polymer matrices to form “mixed matrix membranes” (MMMs) also called hybrid membranes [95,96]. The advantage of using MMMs is that they combine the benefits of the mechanical strength and good processability of polymeric materials with the properties of the filler. According to Kitao et al. depending on the morphology, MMMs can be classified as symmetric or asymmetric membranes. While the first group is characterised by an uniform structure of filler and polymer, the second one is based on a thin and selective filler–polymer layer supported on a nonselective porous substrate [97,98,99].

**Figure 11:**
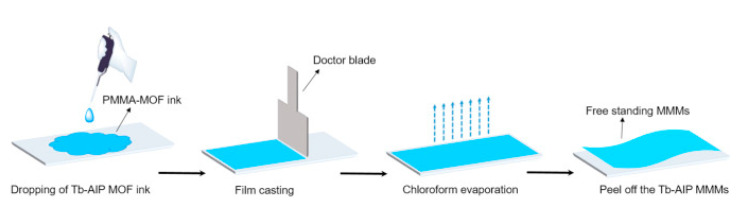
Schematic illustration for the preparation of Tb-AIP mixed matrix membranes (MMMs). Reprinted with permission from [100], Copyright (2017) Elsevier.

The symmetric MMMs preparation method usually consists of the casting of a solution of the filler particles and the polymer mixture on a flat surface, with the help of a knife or doctor blade followed by an evaporation step (Figure 11). In order to avoid particle agglomeration and remove air bubbles stirring or sonication is used before casting. Moreover, it is also very important to control the evaporation rate with low temperature or with partial coverage with the aim to evade the formation of defects. Finally, the excess of solvent will be removed by heating at a certain temperature (depending on the polymer glass transition temperature) under vacuum.

**Figure 12:**
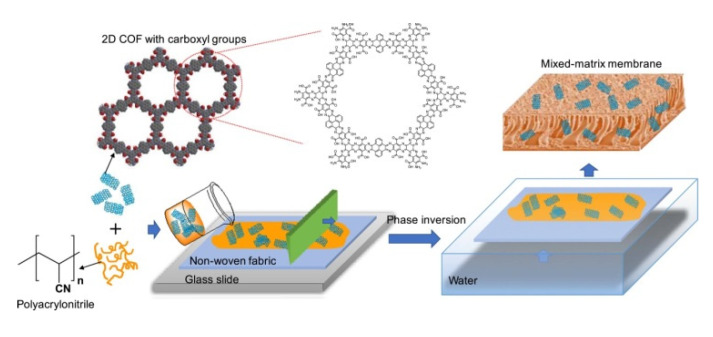
Schematic illustration for the fabrication of mixed-matrix ultrafiltration membrane, molecular structures of the polymer matrix (polyacrylonitrile) and the 2D nanofiller (COF). Reprinted with permission from [99], Copyright (2019) Elsevier.

On the other hand, the asymmetric MMMs preparation method is based on phase inversion via non-solvent induced phase separation (NIPS method) using casting solutions that contain the polymer matrix and the filler (Figure 12) followed by submersing in a non-solvent bath (generally water) at room temperature to exchange the solvent and precipitate the polymer film.

**Figure 13:**
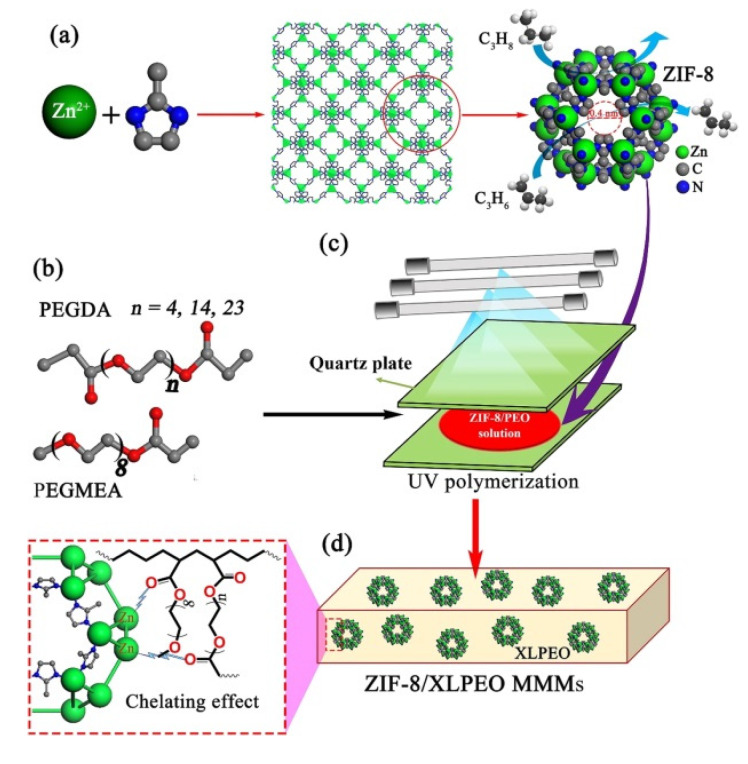
Illustration of fabrication for ZIF-8/XLPEO MMMs: (**a**) ZIF-8 framework synthesised from zinc ion and 2-methylimidazole, (**b**) highly adjustable gas-permeability of XLPEO matrices derived PEGDA and PEGMEA pre-polymers with various molar ratios. (**c**) Formation process of ZIF-8/XLPEO MMMs through the one-step UV polymerisation and (**d**) schematic illustration for interfacial interaction between ZIF-8 fillers and XLPEO matrices in the resulting MMMs. Reprinted with permission from [101], Copyright (2019) Elsevier.

Very recently, Jiang and coworkers reported on the preparation by UV photo-polymerisation of a defect-free MMM based on MOF (Figure 13) [101].

**Figure 14:**
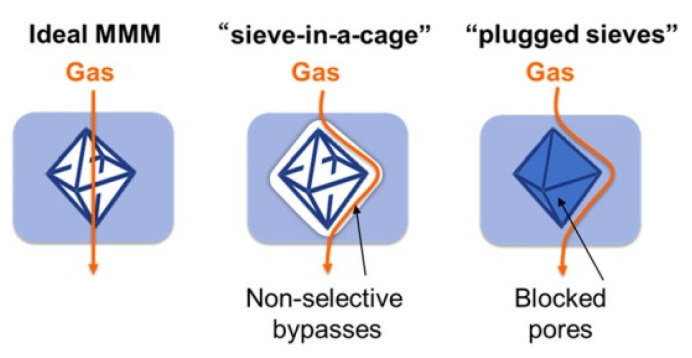
Ideal and nonideal MOF MMM architectures. Reprinted with permission from [118], Copyright (2019) American Chemical Society.

Some of the most common inorganic fillers used in the literature are based on zeolites [102,103,104], carbon molecular sieves, silica [105], metal oxide [106], carbon nanotubes [107,108,109], and graphene oxide (GO) [110,111]. Whereas polyimides (PI) [112,113,114], polysulfone (PSf) [115], polybenzimidazole (PBI) [116,117], and polyacrylonitrile (PAN) [99] have been used as a polymer matrix. An ideal MMM, must be synthesised without suboptimal structures such as “sieve-in-a-cage” or “plugged sieves” (Figure 14) [118,119].

**Figure 15:**
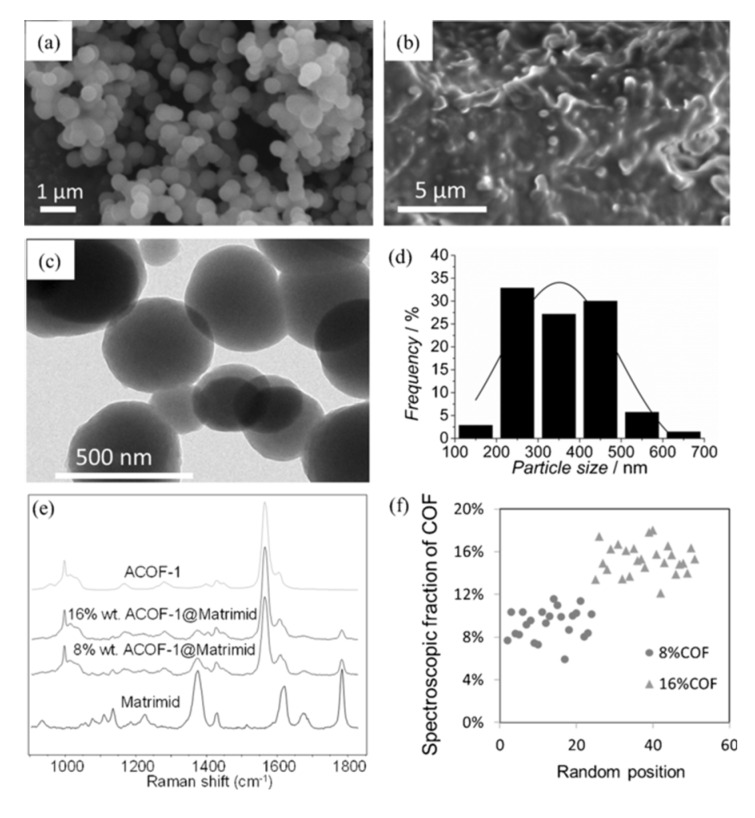
SEM images of (**a**) ACOF-1 and (**b**) the cross-section of a 16 wt % ACOF-1@ Matrimid^®^ MMMs. TEM images (**c**) and particle-size distribution (**d**) of ACOF-1. (**e**) Raman spectra of the pure components and MMMs. (**f**) Spectroscopic fraction of COF calculated at random positions of two different MMMs (8 and 16 wt % COF loading). Reprinted with permission from [127], Copyright (2016) John Wiley and Sons publications.

However, the poor compatibility between the polymer matrices and the inorganic fillers gives rise to non-selective interface void formation, which can affect the MMM performance. In this regard, it has been probed that the organic nature of COFs as well as the presence of organic parts in MOFs enhance the affinity of these materials with the polymers solving, in some cases, the problem of filler agglomeration and precipitation during membrane preparation process. In fact, after the first MOF-based MMM in 2004 [120] numerous types of MOF-based MMMs with diverse pore sizes and structures have been reported for different applications [34,100,121,122,123]. In the case of COFs, it was not until 2016 when Wang and coworkers reported one of the first examples of MMMs based on COFs for CO_2_/H_2_ separation. In this work, a solution of a 2D imine-based COF (COF-LZU1) in poly(vinylamine) (PVAm) was casted onto a PSf supporting membrane [124]. It is highlighted that the presence of strong covalent bonds in COFs enables the preparation of COF-based MMMs under severe conditions. Apart from the filler nature, its loading as well as the election of the correct polymeric matrix are important issues that influence the membrane performance as it was demonstrated by Shan et al. [125]. In a subsequent study, the same group was able to prepare composite membranes for proton conduction from the casting solution of polyvinylidene fluoride (PVDF) and two sulfonated COFs prepared mechanochemically (NUS-9 and NUS-10) [126]. Following a similar fabrication procedure Gascon and coworkers demonstrated the use of bulk microporous azine-linked COF (ACOF-1) for the construction of MMMs using Matrimid^®^ as the polymer matrix. Figure 15 shows the good adhesion between the spherical COF particles and the polymer host [127].

**Figure 16:**
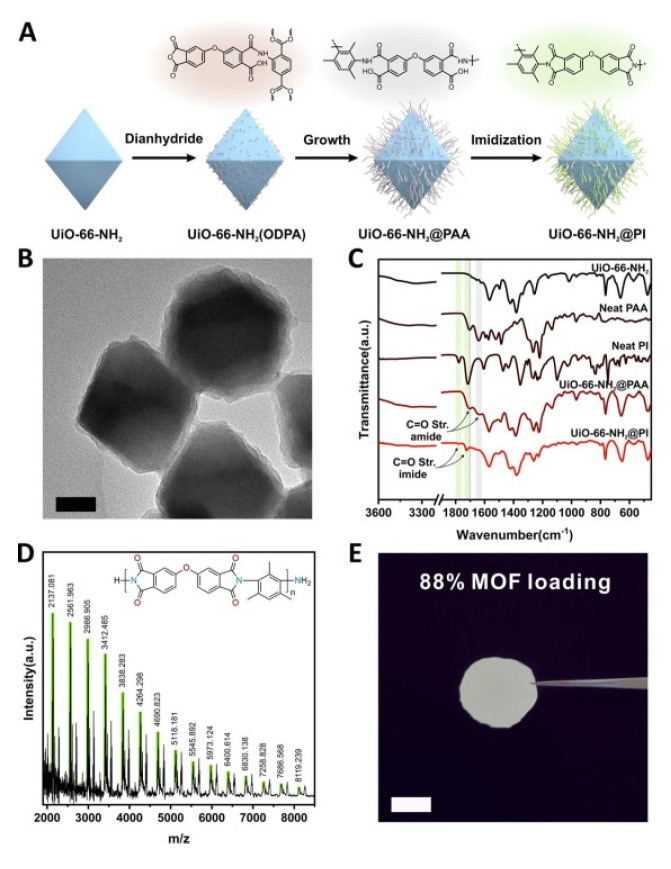
(**A**) Synthetic procedures of UiO-66-NH_2_@PI. (**B**) The TEM image of UiO-66-NH_2_@PI, scale bar 50 nm. (**C**) FT-IR spectra of UiO-66- NH_2_, polymers, and modified UiO-66-NH_2_ and (**D**) the MALDI-TOF spectrum of the digested UiO-66-NH_2_@PI. The peaks highlighted in green correspond to the given polymer structure. (**E**) The photo of a stand-alone single component UiO-66-NH_2_@PI membrane with 88 wt % MOF loading, scale bar 5 mm. Reprinted with permission from [140], Copyright (2018) American Chemical Society.

Another advantage derived from the use of this class of materials as fillers is that MOFs and COFs surface properties can be modified, either by introducing functional groups into the organic ligands or by modifying their surfaces. It is possible to create non-covalent interactions or hydrogen bonds between filler and polymer that increase the affinity with the polymer, favouring the interface compatibility [34,95,128]. Polymer functionalisation strategies have also been used to enhance MOF and COF based MMM performance [129,130]. Some of the approaches used for modifying MOF surfaces are metal or ligand exchange [131,132,133], small molecule surface modification [134,135,136,137], polydopamine modification [128], and grafting polymers onto the MOF surface [138,139]. As an example from the last approach is the strategy developed by Wang et al. based on the covalent grafting of polyimide (PI) brushes to the surface of the MOF UiO-66-NH_2_ (Figure 16). This strategy facilitates the preparation of polymer brush modified MMMs through the interaction (Van der Waals forces) of grafted PI brushes with the PI matrix [140].

**Figure 17:**
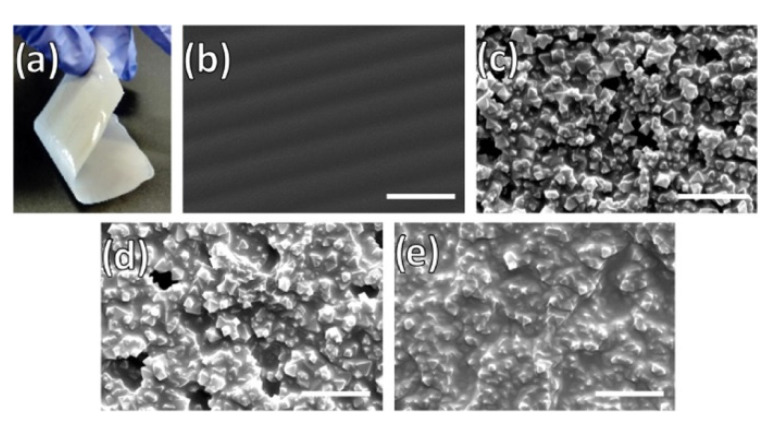
(**a**) Photograph of free-standing 50 wt % MOF-loaded UiO-66-allyl-C MMM. (**b**−**e**) SEM images (top side) of MMMs: (**b**) PDMS-only membrane, (**c**) UiO-66-allyl MMM, (**d**) UiO-66-allyl + PDMS MMM, and (**e**) UiO-66-allyl-C MMM. All MOF-containing MMMs are at 50 wt %. Scale bars are 2 μm. Reprinted with permission from [118], Copyright (2019) American Chemical Society.

A similar approach was taken most recently by Cohen and coworkers who successfully obtained highly stable, flexible, and defect-free MMM with 50 wt % MOF loading, the highest so far achieved. This novel method consists of the covalently grafted MOF particles of UiO-66 with poly(dimethylsiloxane) (PDMS), which increased both the dispersibility and the compatibility between MOF particles and polymer matrix due to the formation of covalent bonds during the preparation of MMMs (Figure 17). This strategy was also used for the fabrication of free-standing thin-film composite membranes with a thickness less than 1 µm [118].

**Figure 18:**
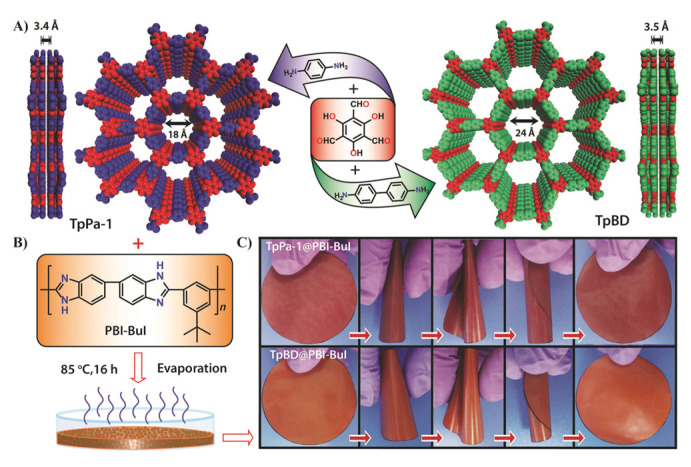
(**A**) Schematic representations of the synthesis of COFs and their packing models indicating the pore aperture and stacking distances. (**B**) Overview of the solution-casting method for COF@PBI-BuI hybrid membrane fabrication. (**C**) Digital photographs showing the flexibility of TpPa-1 and TpBD(50)@PBI-BuI hybrid membranes. Reprinted with permission from [141], Copyright (2016) John Wiley and Sons publications.

**Figure 19:**
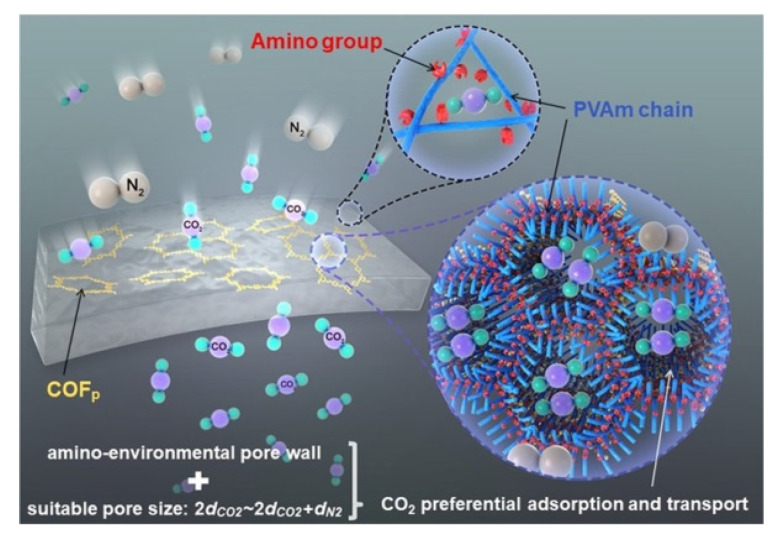
Schematic of COFp-PVAm membranes. Oversized COFp are penetrated by PVAm to create appropriately sized CO_2_-selective channels. d: kinetic diameter. Reprinted with permission from [142], Copyright (2019) American Chemical Society.

In the case of COFs, Biswal et al. showed a methodology to enhance the filler loading in the preparation of flexible and processable COF-polybenzimidazole (TpPa-1@PBI) hybrid membranes (Figure 18) via introducing intermolecular interactions between H-bonded benzimidazole groups of PBI with the COFs. With this approach, they got MMM loading up to 50% in weight [141]. Recently, Wang and coworkers have developed a new methodology based on interface regulation for increasing the compatibility of COF and the polymer matrix. Specifically, this methodology consists of the preparation of MMMs by polymer-COF hybrid materials (COF_p_) obtained through the immobilisation of poly(vinylamine) (PVAm) onto COFs (Figure 19). This approach enhances membrane separation performance due to the modification of the COF pore size as well as the amino environment of the pores [142].

Other decisive parameters to control before the MMM preparation are the size and the morphology of the fillers for avoiding particle aggregation and/or sedimentation [143]. Several reports on MOFs nanosheets [144,145] and nanoparticles [34,98,146,147] have shown how their incorporation into different polymers enhances the performance of MMM significantly. This enhancement is due to a better and closer integration because of the large interfacial areas at the MOF-polymer boundary. Likewise, nanoparticles can be grafted to increase the compatibility with the polymers as well as to enhance the their properties [148].

In the case of COFs, Zhao and coworkers were pioneers in using nanosheets of very stable COFs (NUS-2 and NUS-3) as fillers for the preparation of flexible MMMs. In this case, the good compatibility with the commercial polymers Ultem^®^, a poly(ether imide), and polybenzimidazole (PBI) used, enhanced the surface homogeneity as well as the mechanical properties of the membranes [149]. In a similar approach, 2D TpPa-1 nanosheet clusters (TpPa-1-nc) were dispersed in PEBA and spin-coated onto PVDF supports to prepare membranes used for efficient CO_2_ removal [150]. Liu and coworkers prepared MMMs by the blending method from COF-5 nanosheets obtained sonochemically and Pebax-1657 matrix [151].

Cheng et al. demonstrated a new strategy to increase the interfacial polymer-filler compatibility by the preparation of MOF@COF hybrid fillers. This MOF core coating with COF layers facilitated the formation of hydrogen bonding at the polymer-filler interfaces [152].

The preparation methods for MOFs and COFs membranes were well advanced during the past decade. The proper way to prepare a MOF or COF membrane is highly depending on the intrinsic properties such as structure, crystallization procedure, and compatibility of material and substrate. Additionally the demanding properties for further applications should also be considering (Table 1).

## 3. Properties and Characterisation of MOF and COF Membranes

The following section focuses on the characterisation techniques that allow establishing the properties of the COF and MOF membrane such as stability, flexibility, thickness, permeability, and porosity, with the aim to determine their practical application. As it was mentioned previously, the main advantage of using COF and MOF as a membrane is that their chemical/physical properties can be modified easily. This has mainly been demonstrated by chemical modification of the organic linkers [153,154]. So, membranes could be developed on demand with improved properties for specific applications.

Membranes prepared from MOF and COF material could be characterised at two levels; characterisation of MOF and COF particle/powder and the membranes prepared from the particle/powder. There are several excellent articles focusing on the characterisation of COF and MOF materials [17,155] as well as on the thin films prepared from MOFs and COFs [156,157,158]. Here we will highlight the common techniques used for characterisation of MOF and COF membranes based on fundamental properties such as structural, morphological, textural, and their transport performance.

### 3.1. Characterisation of Structural Properties

Since MOF and COF are crystalline porous materials, it is expected that the membranes based on these materials also show a crystalline structure. In some cases, when the structure of the used MOF and COF in bulk is well-known, the comparison of the patterns of the membrane and the bulk would confirm the membrane structure and indicate the success of the applied method.

The determination of the degree of crystallinity of the membrane is performed by X-ray diffraction (XRD) measurements, which are based on the Bragg Law [159] (Equation (1)) that establishes the relationship between the angular positions of the diffracted beams, the wavelength *λ* of the radiation of the incident X-rays and the interplanetary distances of the crystal planes *dhkl*.

(1) λ=2dhkl sinθ

Apart from the crystallinity, XRD could provide information on the packing mode of the COF and MOF particles forming the membrane as well as the membrane orientation.

For example, Lai et al. used XRD patterns to confirm the crystallinity of TFP-DHF COF film on a support layer and also studied the stacking model of TFP-DHF COF film using XRD patterns and simulations [92]. The similar examples can be found confirming the crystallinity of the COF/MOF nanoparticles and nanosheets embedded in the matrix [160,161].

Small-angle X-ray scattering (SAXS) and wide-angle X-ray scattering (WAXS) can also be used to evaluate the crystallinity or the crystalline phase orientation of such membranes [162,163]. Additionally SAXS can measure the particle size of the nanocrystals, which could be provide useful information to improve the preparation processes [164].

Morphological properties are the most important properties to be determined for membranes since they reveal the surface roughness, continuity, thickness, and are devoid of any pinholes or surface cracks, which can originate preferential ways for the diffusion. As it was mentioned before, it is very important to grow a continuous or defect-free MOF and COF membranes [38].

To gain insight into the internal structure of the membranes, microscopy imaging techniques such as scanning electron microscopy (SEM), field emission electron microscopy (FESEM), transmission electron microscopy (TEM), and atomic force microscopy (AFM) are used. The advantage of these techniques is that it provides direct visual information on the membrane morphology. Cross-sections of COF/MOF membranes analysed under SEM, TEM, or AFM provide information on the membrane thickness and the interface between the COF/MOF layers with the matrix, for example. TEM or FESEM imaging is also used for the obtaining the pore size and shape of the COF and MOF membranes.

However, each microscopic technique has certain resolution and specific sample preparation method with TEM being the most complex. Images obtained from SEM, TEM, and AFM often show comparable results [84,92]. SEM has a resolution of up to 5 nm then, to reach a high resolution high electron-beam energy has to be applied, which can damage the samples. However, SEM could easily observe a relatively large area, which is very important for confirming the continuity of the prepared membrane. FESEM with a resolution of 0.6–0.7 nm or TEM with a resolution of 0.4–0.5 nm are more powerful tools to study the pore size of the micro-mesoporous materials. Atomic force microscopy (AFM) has higher vertical resolution, which makes it a good technique to study the surface roughness [165,166].

Ellipsometry is another interesting technique to analyse surfaces. This optical analysis technique can measure the refractive index of thin films and after the application of mathematical models calculate their thickness [167].

### 3.2. Characterisation of Chemical Properties

Useful techniques for the chemical characterisation of the COF/MOF membranes are Fourier-transform infrared (FTIR) spectroscopy [81,84], X-ray photoelectron spectroscopy (XPS) spectroscopy [168], energy-dispersive X-ray spectroscopy (EDX) [78,169] and Rutherford back scattering spectrometry (RBS) [89].

A FTIR spectrum shows vibration signals of chemical bonds and functions. It is an easy method to confirm the formation of COF/MOF selective layers and study the membrane surface modifications. In some cases, FTIR has been used to study the interface connections between the COF/MOF and matrix due to the presence of specific chemical bonds [87].

XPS measures the binding energy of atoms, which could distinguish the oxidation degrees of elements. XPS can be used for chemical composition analysis and sometimes can be completed and compared with the FTIR analysis [84,92,170]. It is mainly used to analysis the chemistry of the surface providing the elemental composition of the membrane surface.

EDX spectroscopy is usually coupled with SEM or TEM imaging. A quantitative map of elements could be constructed based on the nature and intensity of the interacted X-ray with different atoms and elements present within the detecting region (observation window). The elemental composition could directly be mapped on the SEM or TEM images. EDX is a very useful tool to detect the metal elements of MOF membranes and also different elements present in COF membranes [78,171].

RBS can probe materials by the scattering of an ion beam, which provides the atomic composition and structure information via using modelling software. The elements composition can be determined from the positions of peaks in the energy spectrum. Structure information such as thickness can be determined from the shift position of the peaks [88,89]. It is especially useful for the analysis of a multilayer membrane compare to XPS and EDX, which permit the elemental study on the surface of membrane.

For specific membrane applications the membrane chemical stability is a very important issue to consider. Chemical stability tests are intended to simulate the real operative conditions to which COF and MOF membranes are going to be subjected. For this, the membranes are exposed to different solvents (water and organic solvents) under different conditions (temperature, time, pH, etc.). Indeed, in some cases, it is necessary to carry out a post-synthetic treatment on the membrane with the aim to improve its stability and performance. For example, the membranes can be coated with amphiphilic surfactants after its activation in order to protect them from ambient moisture [172].

### 3.3. Characterisation of Thermal and Mechanical Properties

Other important issues to take into account are the thermal and mechanical stability of the MOF and COF membranes. The thermal stability and flexibility of the membrane can be studied by thermogravimetric analysis (TGA) and differential scanning calorimetry (DSC). TGA is based on measuring the amount and rate of the sample mass changing as a function of temperature or time under a controlled atmosphere. This technique is especially useful for establishing the maximum temperature that the membrane can reach before decomposition. On the other hand, DSC is used to study the difference in the amount of heat required to increase the temperature of a sample and reference as a function of temperature. Thermal transitions of membrane as well as the temperature at which the transition occurs (glass transition temperature, T_g_) could be determined via this technique [173].

The evaluation of the mechanical resistance of the MOF and COF membranes would allow studying the effect of the applied pressure and the mechanical stress on the membrane structure [174]. The common parameters to study are tensile strength, elongation at break, and Young’s modulus.

### 3.4. Textural Characterisation of the Membrane Surface

The Brunauer–Emmett–Teller (BET) surface area was calculated from N_2_ adsorption isotherms measured at 77 K. The experimental data derived from these measurements allowed the determination of the pore size distribution by the application of different models as a function of the pore geometry. The isotherm shape also gives some information about the pore size [87,162,163].

The positron annihilation lifetime spectroscopy (PSA) could also be a powerful technique to characterise the free volume of the membrane. However, it has not been broadly used in the studies of MOF and COF membranes. This technique is based on the principle that the positron annihilation rates through interactions with electrons from different materials are different. The lifetime of positron can be obtained by the emission time of positrons from a radioactive source (for example: ^22^Na, 13 μCi) and detection of the gamma rays released from the positron annihilation. When positrons are injected into a material containing free electrons (such as metals or semiconductors), the implanted positrons will annihilate with the electrons present in the material. For insulators materials such as polymers, MOFs, and COFs implanted positrons interact with electrons in the material to form positronium (Ps), which is a hydrogen-like bound state of an electron and a positron. In case of porous materials, positrons and positronium tend to penetrate and localise in vacancies/voids in the materials and annihilate less rapidly than in the bulk material. For example, in metal simple coulomb attraction forces positrons into electron-decorated vacancies, whereas in insulators the reduced dielectric interaction in a void energetically favours trapping neutral positronium in low-density regions [175]. Ps can exist in two spin states: Para-positronium(p-Ps) that is at a singlet state and Ortho-positronium(o-Ps) that is a triplet state. In molecular materials, o-Ps is easily trapped in the potential well of free volume cavities. So, the lifetime of o-Ps can be converted to the free volume of the material by following Equations (2) and (3):

(2) $$\tau =\frac{1}{2}{\left[1-\frac{r}{r+\Delta r}+\left(\frac{1}{2\pi }\right)sin\left(\frac{2\pi r}{r+\Delta r}\right)\right]}^{-1}$$

(3) $$v=\frac{4\pi }{3}r^{3}I$$

where *τ*, *r*, ∆*r*, and *I* are the o-Ps pickoff lifetime, the radius of the free volume cavities, the thickness of the electron layer, and the intensity of o-Ps [175,176].

Wang et al. applied PSA to determine the free volume of sodium alginate-polyacrylonitrile (SA-PAN) hybrid membrane by incorporation of COF SNW-1 on the surface [177]. Similar examples can be found for obtaining the pore size and distribution for MOF membranes [178,179].

Quartz crystal microbalance (QCM) measures mass changes by measuring the change in frequency of a quartz crystal resonator. QCM can be used under vacuum, in the gas phase and in the liquid phase. For COF and MOF characterisation, QCM can give information on their thickness, porosity and viscoelastic properties of the membrane in liquid media by comparing sorption behaviours of different guest molecules. QCM is a highly effective tool at determining the affinity of molecules uptake to functionalised COF and MOF membrane, which can help the study of transport mechanism of the membrane towards different guest molecules [180,181].

The adsorption/desorption of organic solvent on membranes can change the surface optical characteristics, which could be measured using ellipsometry. This technique coupling ellipsometry and sorption of solvent is called ellipsometric porosimetry, which could provide information on thickness, pore size, and pore size distribution of the COF/MOF layer. However, so far it has not been used for the characterisation of the COF/MOF membrane [182].

### 3.5. Membrane Functional Characterisation

Depending on the final MOF and COF membrane application it will be necessary to carry out additional studies that would predict how they would function under real operative conditions. The following section describes the main parameters determining the potential of MOF and COF membranes for gas and liquid separation as well as for fuel cells. One of the most important factors of membrane performance used in separation processes is the transport capacity, which can usually characterised by two parameters: flux (or permeance) and rejection (or selectivity) [183]. Flux measured from the membrane process is defined as the volume flowing through the membrane per unit area and per unit time, and is usually expressed in terms of L m^−2^h^−1^, while permeance is normalised to the applied pressure, and therefore expressed in terms of L m^−2^h^−1^bar^−1^. Rejection of all the substances could be calculated as a function of the solute concentration in the permeate, *C_i,l_*, and the solute concentration in the retenate (or feed) side, *C_i,0_* Equation (4):

(4) $$Rej_{i}\left(\%\right)=100\left(1-\frac{C_{i,l}}{C_{i,0}}\right)$$

#### 3.5.1. Gas Permeation Capacity

Both defined pore size and functional groups present in the MOF and COF structure make them good candidates for gas separation. In terms of gas permeation and rejection through MOF and COF based membrane, single gases (H_2_, CH_4_, N_2_, and CO_2_) permeability can be obtained by various gas sorption isotherms and then calculated from the rate of pressure increasing (*dp*/*dt*) at a steady state according to Equation (5) [149]:

(5) $$P=\frac{273\times 10^{10}}{760}\frac{VL}{AT\left(\frac{p_{2}\times 76}{14.7}\right)}\left(\frac{dp}{dt}\right)$$

where *P* is the membrane gas permeability in Barrer (1 Barrer = 1 × 10^−10^ cm^3^ (STP) cm cm^−2^ s^−1^ cm Hg^−1^ or 3.348 × 10^−16^ mol mm^−2^ s^−1^ Pa^−1^), V represents the volume of the downstream reservoir (cm^3^), *L* refers to the membrane thickness (cm), *A* is the effective membrane area (cm^2^), *T* is the operating temperature (K), and *p*_2_ indicates the upstream pressure (psia) [149].

The ideal gas selectivity of component *i* over component *j* is calculated on the basis of their different permeability as Equation (6) [149].

(6) $$\alpha_{\left(\frac{i}{j}\right)}=\frac{P_{i}}{P_{j}}$$

In a similar way the mixed gas permeability is calculated from Equation (7) [149]:

(7) $$P=\frac{273\times 10^{10}}{760}\frac{y_{i}VL}{AT\left(\frac{x_{i}p_{2}\times 76}{14.7}\right)}\left(\frac{dp}{dt}\right)$$

where the *x_i_* and *y_i_* represent the molar fractions of component *i* in both up- and down-stream.

The mixed gas separation factor is calculated on the basis of Equation (8) [149]:

(8) $$S_{\left(\frac{i}{j}\right)}=\frac{y_{i}/y_{j}}{x_{i}/x_{j}}$$

The permeability or selectivity for the same gas pair based on single gas and mixed gas are usually different owing to the competitive adsorption and diffusion of the mixed gas in the membrane [149]. The selectivity defined in membrane-based separation emphasises the difference of gas permeability, which is how fast the gas can penetrate through the membranes. In membrane based gas separation can be typically described using the solution−diffusion model, in which permeability *P* can be expressed by Equation (9):

*P* = *S* × *D* (9)

where *S* is the solubility of a particular gas in polymer and *D* is the diffusivity of that specific gas [149]. The gas solubility is controlled by the affinity of gas toward polymer membrane surface and diffusivity depends on the relative size of the gas molecular to the pore size of the framework. In case of MOF and COF mixed matrix membranes, adding fillers (MOF or COF) into polymers can affect both *S* and *D*, while the affected trend depends on the filler’s properties, loading and other factors as well.

To compare the membrane performance with the existing materials, there is a well-known trade-off relationship between permeability and selectivity, as originally reported Robeson in 1991 (revised in 2008) [184,185].

#### 3.5.2. Liquid Permeation and Rejection Capacity

Liquid transport capacity of the membrane is a very important factor for several applications. In an aqueous media process, hydrophilic membranes can facilitate the water transport, increase the process efficiency and reduce the cost. Similarly, for non-aqueous solvents a hydrophobic membrane is required. Usually, the permeation properties for a liquid are determined by passing protic and aprotic organic solvents (acetonitrile, water, ethanol, and methanol) through the membrane under specific conditions such as concentration, temperature, thickness, pressure, and/or electric field. The membrane surface hydrophilicity is usually tested by contact angle measurements. The contact angle of a liquid drop on an ideal solid surface refers to the mechanical equilibrium of the drop under the action of three interfacial tensions Equation (10), as was described by Thomas Young in 1805 [186].

*γlvcosθY* = *γsv* − *γsl* (10)

where *γlv*, *γsv*, and *γsl* represent the liquid–vapour, solid–vapour, and solid–liquid interfacial tensions, and *θY* is the Young’s contact angle or static contact angle. By Young’s equation, small contact angles (≤90°) correspond to high wettability, which means the membrane surface is hydrophilic. In the contrast, while large contact angles (≥90°) correspond to low wettability, which means the membrane surface is hydrophobic. However, in practice, there exist many metastable states of a droplet on a solid, and the observed contact angles are usually not equal to *θY*. If the three-phase contact line is in actual motion, the contact angle produced is called a “dynamic” contact angle. At a low measuring speed, dynamic contact angle could be close or equal to a properly measured static contact angle [187]. Solvent transport capacity can also be directly measured by a dead-end filtration system [92]. The transport capacity defined with solvent flux (*J*) and permeance (*P*) are calculated by Equations (11) and (12):

*J* = *V/At* (11)

*P* = *J/∆p* (12)

where *V* is the collected solvent volume (*L*) across the membrane during a time period of t (h), A is the effective membrane area (m^2^), and Δ*p* is the trans-membrane pressure drop (bar) [92].

In case of liquid separation, especially dehydration of the water–alcohol mixture through pervaporation is well studied in polymer-MOF or COF mixed matrix membranes [84,168]. The permeation flux (*J*, g m^−2^h^−1^), separation factor (*α*), and pervaporation separation index (PSI) are calculated from Equations (13)–(15):

*J* = *Q/At* (13)

(14) α=PWFWPAFA

*PSI* = *J (α − 1)* (15)

where *Q* (g) is the weight of permeate through the membrane area *A* (m^2^) under the time interval t (h) for permeate collection, and *P* and *F* are mass fractions of water (*W*) or alcohol (*A*) in the permeate and feed solution, respectively [84]. Incorporation of MOFs and COFs with hydrophilicity groups can enhance the hydrophilicity of the membrane, which increases the water flux during the membrane process. In addition, ordered pore size, good compatibility, and multi-functionality of MOFs and COFs can also improve both the membrane permeability and the selectivity facing different liquid mixture.

#### 3.5.3. Solute Permeation and Rejection Capacity

There are two main types of solute usually studied in membrane applications: salts and organic compounds. The alts’ rejection depends on pore size, membrane surface charge, and the ions present in the solute. Larger pore size leads to higher permeation and lower selectivity towards different ions. However, ions with the same charge as the applied membrane and multivalent ions show stronger repulsion through the membrane. By these principles, membranes with the same charge as the targeting ions have a relatively high rejection and good antifouling properties. For example, Wu et al. have prepared a thin-film nanocomposite (TFN) membrane incorporating COF (SNW-1) into the polyamide (PA) layer on a polyether sulfone (PES) substrate (PA-SNW-1/PES). Due to the PA layer that is negatively charged the TFN membrane shows a stronger repulsion towards di-anionic anions (SO_4_^2−^) than monovalent anions (Cl^−^) [87]. The concentration of the salt solution was used to detect the conductivity of the solution using an electrical conductivity meter. The salt rejection was then calculated using Equation (4).

As for organic compounds, dyes are usually used as the model compound to study the rejection properties of MOF and COF membrane towards organic compounds. There are various different types of dyes with different solubility (water soluble or solvent soluble) and functional groups that would be used for this purpose. Dye rejection depends mostly on the membrane pore size. The dye rejection measurements are conducted in water or in organic solvents and the concentration of dyes are usually measured by UV-vis spectroscopy and the rejection is calculated using Equation (4) [92,188].

#### 3.5.4. Proton Conductivity Properties

COF and MOF membranes have also been used as a solid electrolyte in fuel cells. Such membranes must have good proton conductivity. For this purpose, ionic conductivity measurements are performed on MOF/COF pellets using electrochemical impedance spectroscopy [126,189,190,191,192]. Conductivity values are calculated from equation σ = L/(R × A), where σ is the conductivity value (S cm^−1^), L the thickness of the sample (cm), A is the electrode area (cm^2^), and R (Ω) is the electrolyte resistance corresponding to the real Z′ Nyquist plot. In some cases, the conductivity values measured at different temperatures are correlated by means of an Arrhenius type equation that allows obtaining the activation energy (E_a_) of the system, which will establish the type of mechanism that dominates the conduction either by Grotthuss (≤0.4 eV) or Vehicular (>0.4 eV) [193]. The proton conductivity and activation energy could be improved by optimising the interfacial interactions [189,190], introducing functions that provide additional proton-transport sites [191].

## 4. Applications of MOF and COF Membranes

Membranes are widely used in processes engineering since they are often technically simpler and more energy efficient than conventional separation techniques. Particularly, they are used on a large scale to produce potable water from sea and brackish water, to clean industrial effluents and recover valuable constituents, to concentrate, purify, or fractionate macromolecular mixtures in the food and drug industries, and to separate gases and vapours in petrochemical processes [165]. Among all the varieties of membranes that exist in the market, the ability of MOFs and COFs to easily control their pore size and shape as well as their properties, makes them ideal candidates for different membrane applications [29,35,38,194,195]. Here, we will focus on some of the most recent progress made in MOF and COF membranes for gas separation, liquid separation, and fuel cells.

### 4.1. Gas Separation

Since the discovery of MOFs and COFs, one of their main applications was related to the capture and separation of gases of industrial and environmental interest. Later on, their processing in the form of membranes, to be tested under real operating conditions, also gave rise to very interesting results in CO_2_ recovery, H_2_ purification, and hydrocarbon separation among others, as it has been already discussed in previous reviews [31,196,197,198,199,200,201,202]. Zeolitic imidazolate frameworks (ZIFs) are often used in the preparation of membranes for gas separation owing to their high thermal and chemical stability [114]. However, the separation of small gas molecules by the use of continuous COF membranes is quite challenging due to the characteristic pore size of these materials.

#### 4.1.1. CO_2_ Recovery

Carbon dioxide is one of the main gases emitted by thermal power plants along with N_2_, O_2_, H_2_O, and its separation before its release into the atmosphere, especially from N_2_, is very important since it is implicated in the greenhouse effect. Likewise, CO_2_ separation from CH_4_ in natural gas purification is also very important for avoiding pipeline corrosion among other issues. Usually, polymeric membranes offer an impact solution to these separations, replacing other solutions like distillation, which is highly energy consuming. However, CO_2_ can produce polymer plasticisation; it is changes of the polymeric structure that makes the membrane unusable. It is important to highlight that due to the volume of the gas mixture to treat in CO_2_/N_2_ and CO_2_/CH_4_ separations, permeability in membranes is preferred than selectivity as well as strong interactions with CO_2_ molecules. To meet the last requirement, the rational design of MOF and COF structures allows incorporating functional groups with high affinity to CO_2_ such as, –NH_2_, –OH, and –COOH that will improve the CO_2_ adsorption properties. Taking this strategy into account, Lin and coworkers demonstrated the beneficial effect caused by the post-synthetic modification with 3-isocyanatopropyltriethoxysilane of the CAU-1 MOF membrane decorated with amino groups, which gave rise to relatively high permeabilities as well as increasing the CO_2_/CH_4_ selectivity values [203].

**Figure 20:**
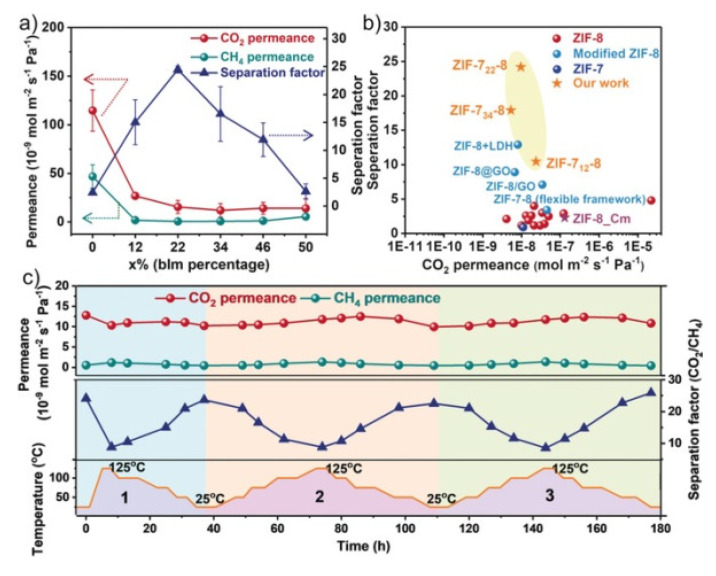
(**a**) CO_2_/CH_4_ separation performance of the mixed-linker ZIF-7_x_-8 membranes. The ZIF-7_22_-8 membrane shows the highest CO_2_/CH_4_ separation factor of 25. (**b**) Comparison of CO_2_/CH_4_ separation performance of our ZIF-7_x_-8 membranes with other ZIF membranes. (**c**) Temperature swing stability of the ZIF-7_22_-8 membrane for CO_2_/CH_4_ separation. Reprinted with permission from [205], Copyright (2019) John Wiley and Sons publications.

The separation of CO_2_ through the use of MOF membranes has been widely reported in many reviews [34,204,205,206,207]. Usually, the pore size of these structures plays an important role as molecular sieves. For example, one of the most interesting ZIFs to carry out the CO_2_/CH_4_ and CO_2_/N_2_ separations by molecular sieving is ZIF-8 since its pore aperture is 0.34 nm and shows high thermal and chemical stability. However, its flexible lattice originates the low separation factors. Wang and coworkers reported an excellent mixed linker strategy for the preparation of ZIF-7_x_-8 membrane (440–600 nm) with high separation efficiencies due to the decrease in pore size. Particularly, the maximum separation factors obtained for CO_2_/CH_4_, H_2_/CH_4_, and CO_2_/N_2_ were 25, 17, and 20, respectively, higher than other ZIF-8 membranes reported. Moreover, the performance is maintained after 180 h (Figure 20) [205].

**Figure 21:**
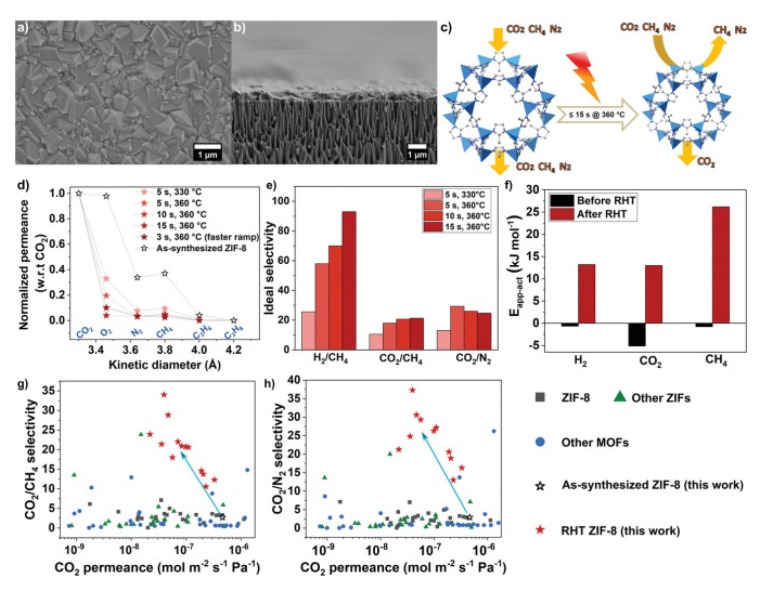
Scanning electron microscopy images of the as-synthesised ZIF-8 membrane: (**a**) top view and (**b**) cross-sectional view. (**c**) Schematic representation of the rapid heat treatment (RHT) process. (**d**–**h**) Gas separation properties of RHT ZIF-8 membranes: (**d**) gas separation characteristics of ZIF-8 at 30 °C as a function of rapid heat treatment parameters. (**e**) Ideal selectivity for various gas pairs at 30 °C as a function of the dwell time and temperature. (**f**) The calculated apparent activation energy for ZIF-8 membrane before and after RHT. Comparison of the CO_2_ separation performance of RHT ZIF-8 membranes with other reported MOF membranes: (**g**) CO_2_/CH_4_ and (**h**) CO_2_/N_2_, arrow shows the improvement in the separation performance after RHT. Reprinted with permission from [208], Copyright (2019) John Wiley and Sons publications.

Very recently, Babu et al. reported another approach to limit the lattice flexibility in ZIF-8 by carrying out a post synthetic rapid heat treatment (RHT) on the ZIF-8 membrane, which lead to an increase of the lattice stiffness as well as the highest CO_2_/CH_4_ and CO_2_/N_2_ selectivity reported so far (Figure 21) [208]. This MOF was also used by Caro and coworkers for the preparation of a ZIF-8-ZnAl-NO_3_ layered double hydroxide (LDH) composite membrane on the γ-Al_2_O_3_ support that showed and the CO_2_ permeance is 0.0977 × 10^−7^ mol m^−2^s^−1^Pa^−1^ and the separation factor for CO_2_/CH_4_ of 12.9 that exceeded the corresponding Knudsen values (0.6) due to the affinity of LDH for CO_2_ [209]. ZIF-7 that presents a pore size about 0.3 nm has also been used for this application. For example, Coronas and coworkers fabricated by microfluidic a ZIF-7 membrane on the inner face of a polysulfone (PSF) hollow fibre with the highest separation factors of CO_2_/N_2_ and CO_2_/CH_4_ obtained for this ZIF [210].

Caro et al. have developed a 2D COF membrane based on ACOF-1 and alumina by solvothermal synthesis. ACOF-1 on the surface of alumina support provides a pore size of about 0.94 nm and abundant polar groups on the pore wall, which are good to enhance the CO_2_ capacity and CO_2_/CH_4_ selectivity. The experiment results show the membrane provided a high selectivity for the CO_2_/CH_4_ gas pair with a reasonable CO_2_ permeance. The overall performance surpasses the Robeson upper bound (2008) shows better performance than existing materials [211].

Apart from the recent reports based on continuous ZIF membranes, the majority of MOF and COF membranes are based on MMM since they combine the properties of crystalline porous materials and polymers. However, as it was mentioned in Section 2.4, a high compatibility and dispersity between both materials is needed to get gas separation performances and avoid membrane defects like pinholes or cracks. The filler properties such as morphology, size, orientation as well as the type of polymer matrix used will have important consequences on the permeability and selectivity values. Zheng et al. studied the influence of ZIF-8 particles size (40, 60, 90, and 110 nm) obtained by the microemulsion method at different loadings (from 0 to 20 wt %) in the preparation of Pebax based MMMs. They found that the CO_2_ permeability increased with the filler loading as well as for the particle size owing to the increase in free volume and surface area. The best results were obtained for the MMM loads with 5 wt % of ZIF-8 and a particle size of 90 nm, which showed a CO_2_ permeability of 99.7 Barrer and CO_2_/N_2_ selectivity of 59.6 [212]. In another approach, Lai et al. applied thermal annealing treatment to ZIF-8 particles in order to modify their structure by introducing little defects before their integration into a poly(styrene-co-butadiene) (SBC) polymer matrix. The results showed an increase of the CO_2_ permeability and CO_2_/N_2_ selectivity in comparison with the naked SBC membrane due to the removal of host molecules and the breaking off the Zn‒N bond in the annealed ZIF-8 framework at 300 °C [213]. Very recently, Mandal and coworkers studied the CO_2_/N_2_ separation performance of ZIF-8 nanoparticles (100 nm) dispersed into a poly(vinyl alcohol) (PVA)/piperazine glycinate (PG) polymeric matrix and showed that for 5 wt % ZIF-8 loadings the CO_2_ permeance (82 GPU) and CO_2_/N_2_ selectivity (370) of the resulting MMM increased 82.2% and 76.2%, respectively, compared to the bare PVA/PGG membrane. These results exceeded the Robeson’s upper bound [214]. Yang and coworkers reported ZIF-8/P84 MMMs with a CO_2_ permeability of 10.92 Barrers with CO_2_/CH_4_ separation factor of 92.6, assuming an improvement of 339% and 35.7% with respect to the pure polymer. These values also surpass the Robeson’s upper bound [215]. Bae and coworkers studied the CO_2_/CH_4_ separation performance of a MMM that integrated 2D (ns-CuBDC) and 3D (ZIF-8) MOFs as a filler. The combination of both MOFs gave rise to an improvement in the CO_2_/CH_4_ selectivity as well as CO_2_ permeability [216]. The use of a heterogeneous mixture of two different polymer matrixes for the preparation of MMM has also been very recently reported by Coronas and coworkers. They demonstrated the positive effect on the CO_2_/CH_4_ and CO_2_/N_2_ separation performance by the incorporation of 10 wt % ZIF-8 nanoparticles in a heterogeneous blend of the highly permeable polymers 6FDA-DAM and PIM-1 (polymer of intrinsic microporosity, PIM) [217]. The preparation of MMMs based on PIM-1 to improve both its selectivity and its permeability has been widely studied also by other authors. For example, Khdhayyer et al. studied PIM-1 based MMMs prepared from the incorporation of different types of MIL-101 MOF: MIL-101 (particle size ca. 0.2 µm), NanoMIL-101 (particle size ca. 50 nm), ED- MIL-101 (MIL-101 functionalized with ethylene diamine), and NH_2_-MIL-101 (MIL-101 synthesised using 2-aminoterephthalic acid). The best results were obtained for MIL-101, which, maintaining the excellent ideal CO_2_/N_2_ and CO_2_/CH_4_ selectivity of the bare polymer enhanced the CO_2_ permeability reaching a value of 35.600 Barrer for 47 vol. % MIL-101. Apart from the PIM-1/MIL-101, PIM-1/nanoMIL-101 also surpass the separation trade-off defined by Robeson [218].

**Figure 22:**
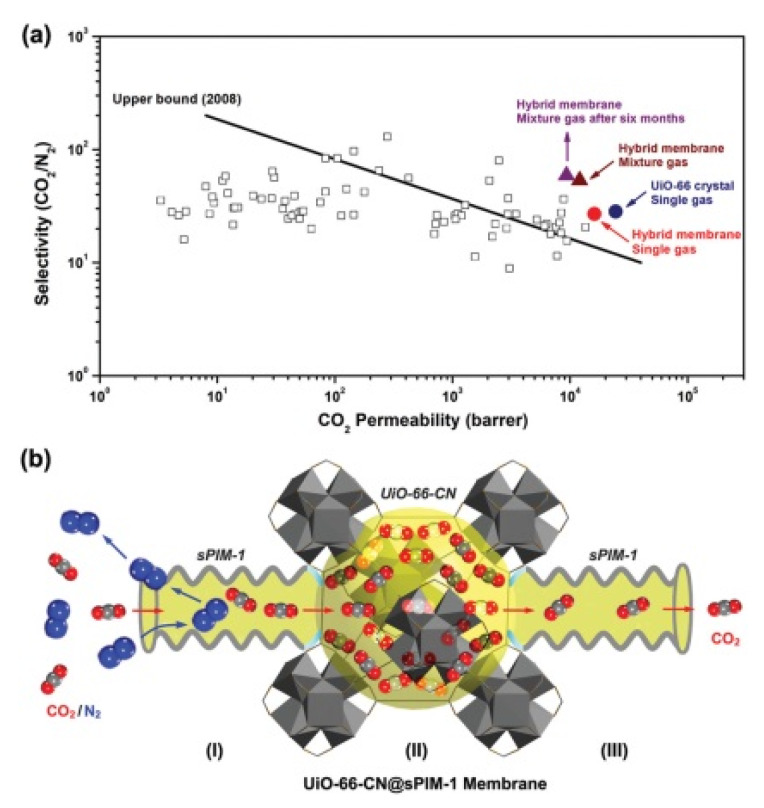
A graphic illustration of CO_2_ and N_2_ mixture gas across the UiO-66-CN@sPIM-1 membrane. (**a**) A summary of MOF-based hybrid membranes reported up to date for the application in CO_2_/N_2_ separation (**b**) a schematic illustration of CO_2_ and N_2_ transport across the UiO-66-CN@sPIM-1 membrane. Reprinted with permission from [222], Copyright (2019) John Wiley and Sons publications.

The excellent stability of zirconium MOFs as well as their suitable pore size (MOF-801 [219], Bipyridine-based UiO-67 [220], UiO-66-NH_2_ on@GO [221]) makes them ideal candidates for the preparation of MMM based on different polymer matrixes always leading to improvements in gas separation performances in comparison to the bare polymer. In this context, very recently, Yu et al. reported an excellent methodology to build a CO_2_ free-ways MMMs based on different loadings of UiO-66-CN linked covalently to PIM-1 and subjected to a heat treatment. The resulting UiO-66-CN@sPIM-1 MMM showed exceptionally high CO_2_ permeability values (15,433.4 and 22,665 Barrer), which surpass the upper-bound of Robeson as well as long-term stability (Figure 22) [222].

Aiming to improve the filler–polymer compatibility, Cohen and coworkers grafted post-synthetically hydride-terminated poly(dimethylsiloxane) (PDMS; <5 wt %) to UiO-66 particles, which gave rise to free-standing and defect-free 50 wt % MOF-loaded MMMs with CO_2_ permeabilities higher than PDMS membranes [118]. In similar work, Huang and coworkers grafted imidazole-2-carbaldehyde (ICA) to UiO-66-NH_2_ before its incorporation to the Matrimid polymer matrix. In this case, the presence of ICA apart from a decrease in the pore size of the MOF improved 40% the CO_2_/CH_4_ selectivity of UiO-66-NH_2_@ICA/Matrimid^®^ with respect to UiO-66-NH_2_/Matrimid^®^ MMM [223]. Chen et al. reported an innovative approach consisting of the preparation of MOF@COF hybrid materials (UiO-66-NH_2_ cores covert by TpPa-1 layers) to be used as fillers in the preparation of MMMs. The good polymer-filler compatibility as well as the size-selective pores in MOFs gave rise to improvements of 48% and 79% in both CO_2_ permeability and CO_2_/CH_4_ selectivity, respectively [152].

Selection of the MOF and the polymer matrix has important influence in the gas separation performance. Taking this into account, Sabetghadam et al. prepared eight MMMs with four different MOFs at 25 wt % loadings (NH_2_-MIL-53(Al), MIL-69(Al), MIL-96(Al), and ZIF-94) and two different polymer matrixes (6FDA-DAM and Pebax) that were tested for CO_2_/N_2_ separation. All MMMs exceeded the Robeson upper-bound limit due to an enhancement in CO_2_ solubility that caused improvements in both the selectivity and the permeability of this gas, obtaining the best results for MIL-96(Al) [224].

**Figure 23:**
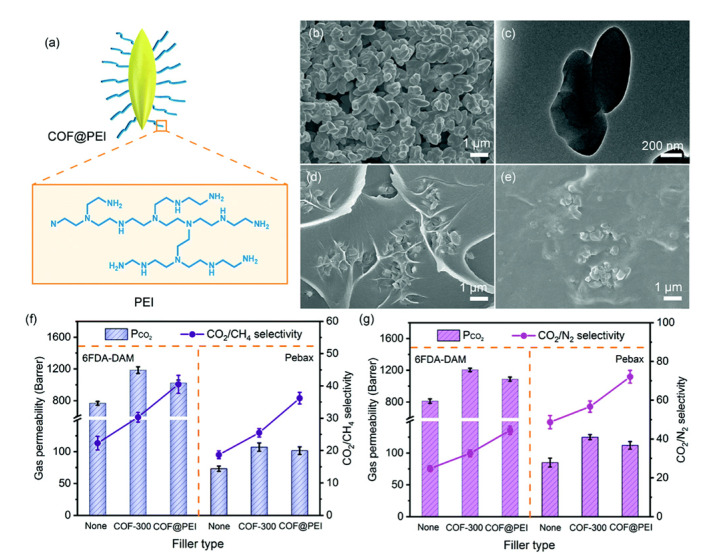
(**a**) Illustration of the PEI grafting on the surface of COF-300 in COF@PEI. (**b**) FESEM and (**c**) TEM images of COF@PEI particles. Membrane cross-sectional FESEM images of (**d**) COF@PEI/6FDA-DAM-7 and (**e**) COF@PEI/Pebax-10 MMMs. (**f**) CO_2_/CH_4_ and (**g**) CO_2_/N_2_ separation performance of pure polymeric membranes and MMMs containing different fillers. Reprinted with permission from [226], The Royal Society of Chemistry, Copyright (2019).

In the case of COF MMM, Kang et al. dispersed two water stable 2D COFs (NUS-2 and NUS-3) into poly(ether imide) (Ultem^®^) and polybenzimidazole (PBI) polymer matrixes. In the case of NUS-2@Ultem and NUS-3@Ultem reached selectivity for CO_2_/CH_4_ of 33 and 30, respectively with low CO_2_ permeability (10 and 15 Barrer, respectively) [149]. Karhul and coworkers incorporated chemically stable isoreticular COFs (TpPa-1 and TpBD) to the polymer (PBI-BuI) matrix. The resulting MMMs exhibited H_2_, N_2_, CO_2_, and CH_4_ permeability values up to 7 times higher than for the bare polymer while maintaining CO_2_/N_2_ and CO_2_/CH_4_ separation factors [141]. Gascon and coworkers evaluated the beneficial effect of the presence of azine-linked COF (ACOF-1) in the polymer matrix Matrimid (ACOF-1@Matrimid) for the separation of CO_2_ from equimolar mixtures of CO_2_/CH_4_. Particularly, at 16 wt % ACOF-1 loading the CO_2_ permeability were more than double the original Matrimid^®^ polymer and the CO_2_/CH_4_ separation factors considerably higher due to both fast transport of gases and CO_2_-philic properties of the filler [127]. The same group demonstrated the importance in the election of the filler–polymeric matrix pair by the preparation of MMMs for CO_2_/N_2_ separation with three different polymers and the ACOF-1 COF at different loadings. The best result was obtained for the MMM based on Matrimid^®^ and 16 wt % ACOF-1 loading, which showed a selectivity increase from 29 to 35, as well as an enhancement in permeability from 9.5 to 17.7 Barrer [125]. Zou et al. reported a PEBA based MMM with 1 wt % of the 2D COF structure with amide groups that showed one of the highest separation factor (72) for equimolar CO_2_/N_2_ mixture consequence of competitive adsorption [150]. Duan et al. also incorporated 0.4 wt % of COF-5 nanosheets, obtained by a sonochemical method, into the Pebax-1657 matrix, which resulted in an increase in CO_2_ permeability (493 Barrer) and CO_2_/N_2_ selectivity (31.3) with respect to the bare polymer [151]. Jiang and coworkers managed to improve the low selectivity that PIM-1 (polymer of intrinsic microporosity, PIM) presents to CO_2_ by introducing SNW-1 COF into their pores in a 27.4% and 37.6% for CO_2_/CH_4_ and CO_2_/N_2_, respectively [225]. Recently, Cao et al. designed CO_2_-selective pores through the immobilisation of poly(vinylamine) (PVAm) inside the pores of a 2D COF. Then, this hybrid material (COF_p_) was dispersed in the PVAm polymer matrix for the preparation of MMMs, which showed membrane performance for CO_2_/N_2_ and CO_2_/CH_4_ above the Robeson upper bound due to the amino-environmental pore wall and to the decrease in pore size after the adsorption of CO_2_ molecules. Moreover, these authors have been pioneers in establishing a one-dimensional model for the transport of gas molecules [142]. Very recently, Zhao and coworkers reported the integration of a 3D COF (COF-300) in two different polymer matrixes, exhibiting in both cases an increase of more than 50% CO_2_ permeability and improvements in CO_2_/CH_4_ and CO_2_/N_2_ selectivity owing to the high surface area and ultra-small pores (4 Å) of the porous filler. Moreover, they carried out the grafting of COF-300 with polyethylenimine (PEI) before blending, with the aim to increase the polymer/COF compatibility by H-bonding formation and enhanced the CO_2_-philicity as well as polymer affinity (Figure 23). The results showed enhancements in CO_2_ permeability and CO_2_/CH_4_ selectivity in comparison to the bare polymer [226].

#### 4.1.2. H_2_ Purification and Recovery

Hydrogen, considered a clean and renewable fuel, is usually obtained from the steam-methane reforming (SMR) process followed by water-gas-shift (WGS). This process involves the catalytic oxidation of methane through water vapour to give rise to a gas mixture that contains mainly hydrogen and carbon dioxide as well as unreacted methane, and carbon monoxide. The purification of the hydrogen from the rest of the products is highly demanded for commercialisation reasons. Likewise, H_2_/CO_2_ gas separation is highly important in the CO_2_ capture from the precombustion process in power plants. In this regard, the use of H_2_ or CO_2_ selective membranes offer an energy efficient and ecofriendly solution for the separation of CO_2_/H_2_ mixtures and achieve high purity levels of H_2_ [227].

**Figure 24:**
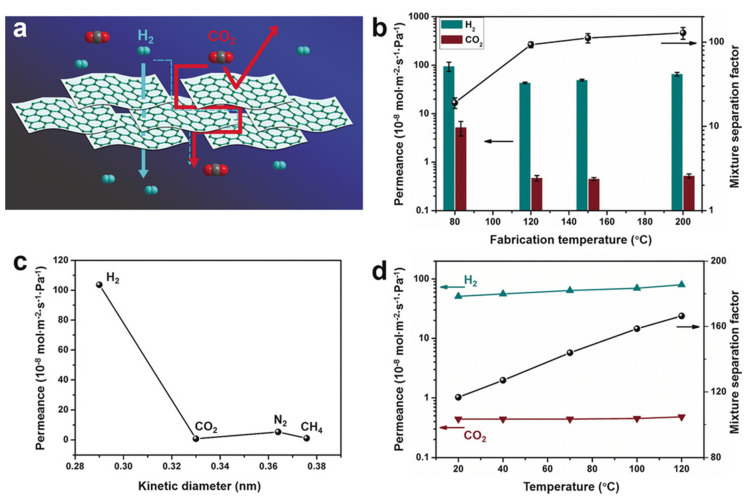
(**a**) Illustration of the hypothesis of gas separation through porous Zn_2_(Bim)_3_ nanosheets. Only Zn atoms are shown for clarity, and the light blue planes represent the nanosheets regardless of their amphiprotic natures. The solid and dashed lines represent the pathways of H_2_ (blue) and CO_2_ (red). (**b**) Binary gas separation performance of equimolar H_2_/CO_2_ through the Zn_2_(Bim)_3_ nanosheet membranes prepared at different temperatures via the hot-drop coating method. (**c**) Single gas permeation through a Zn_2_(Bim)_3_ nanosheet membrane prepared at 200 °C. (**d**) Effect of varying temperature on H_2_/CO_2_ permeance and mixture SF of a Zn_2_(Bim)_3_ nanosheet membrane prepared at 150 °C. Reprinted with permission from [230], Copyright (2017) John Wiley and Sons publications.

As it was mentioned before, one of the requirements to get excellent separation performance is using ultrathin membranes. In this context, Hou et al. reported the formation of ZIF-8 over APTES- titatina-modified PVDF hollow-fibre membranes with excellent H_2_ permeance (up to 201 × 10^−7^ mol m^−2^s^−1^Pa^−1^) and ideal H_2_/CO_2_ selectivities [228]. The use of membranes based on MOF nanosheets have been widely used in H_2_/CO_2_ gas separation processes since the first report from Yang and coworkers in 2014 [229]. Later on, the same group reported the preparation of sub-10 nm-thick ultrathin membranes based on 2D MOF nano sheets obtained by a novel soft-physical exfoliation strategy (Figure 24). The membrane thickness as well as the size-exclusion influence of the MOF membranes lead to H_2_ permeance of up to 8 × 10^−7^ mol m^−2^s^−1^Pa^−1^ at high temperatures and a H_2_/CO_2_ separation factor of 166 [230].

Zhang and coworkers were pioneers in the preparation of highly oriented, continuous, and scalable tubular ZIF nanosheet membranes for H_2_/CO_2_ separation using different strategies. In one case, they carried out the ZnO self-condensation by the help of ammonia that acted as a modulator during the direct growth of the membrane. They got to prepare a 50 nm-thickness nanosheet membrane that showed a permeance of H_2_ of 2.04 × 10^−7^ mol m^−2^s^−1^Pa^−1^ and ideal selectivity of 53, 67, and 90 for H_2_/CO_2_, H_2_/N_2_, and H_2_/CH_4_, respectively [231]. In a subsequent report, the presence of GO during the in-situ growth strategy helped to control the orientation growth, obtaining a 200 nm-thickness membrane, which showed excellent H_2_/CO_2_ ideal separation selectivity of 106 with a H_2_ permeance of 1.5 × 10^−7^ mol m^−2^s^−1^Pa^−1^ [232]. Very recently, the same group achieved for the first time to synthesise Co-based ZIF nanosheets membrane with a thickness of ca. 57 nm, which reached H_2_/CO_2_ selectivity values as high as 58.7 and a H_2_ permeance of 17.2 × 10^−8^ mol m^−2^s^−1^Pa^−1^. In this case, the applied method, based on ligand vapour-phase transformation, allowed controlling the thickness as well as the orientation of the nanosheets during the membrane growth since both issues affect gas separation performance [75]. In a different report, Coronas and coworkers reported preparation of double ZIF membranes inside polyimide P84 hollow fibres. On the inner surface of the hollow fibres, the ZIF-9 layer was firstly crystallised using liquid phase epitaxial (LPE) synthesis with a microfluidic system. Then ZIF-67 or ZIF-8 layer was coated on the ZIF-9 layer by the same method in order to reduce the CO_2_ adsorption on the ZIF-9 surface. The results show a significant improvement of H_2_ selectivity and permeance [233].

Liu and coworkers demonstrated how the preparation of highly *c*-oriented NH_2_-MIL-125(Ti) membranes resulted in H_2_/CO_2_ selectivity values six times higher than those randomly oriented counterparts due to the elimination of grain defects as well as to the decrease of diffusion barriers [57].

In the case of MOF based MMMs, very recently, Urban and coworkers reported the preparation under mild conditions of MMMs based on UiO-66-NH_2_ and a flexible polymer, which showed excellent H_2_ separation properties caused by the formation of H-bonding between the amino and carboxylic groups from the constituent materials. The exceptional compatibility between them, allowed obtaining membranes with MOF loads up to 55 wt % and CO_2_ and H_2_ permeability of 2494 and 2932 Barrers, respectively. It is a highlight that the gas permeability performance increased 16-fold with respect to the bare polymer and it is maintained over 5300 h of operation at ambient conditions [234].

Very recently Chen et al. prepared tubular MMMs based on MOFs-polymer-tubular ceramic support. In this study, MOFs (NH_2_-GAU-1 and NH_2_-MIL-53) nanoparticles were dispersed in solvent with addition of PMMA. Then the mixtures were coated on the organosilica (BTESE) modified ceramic support to form the MMMs. One of the resulting MMMs with 20% NH_2_-MIL-53 and 80% PMMA loading provide a high H_2_/CO_2_ separation factor of 53.1, which is higher than most reported MMMs and higher than the Robeson upper bound limit of gas separation. These results show the high potential application of the MOF-polymer MMMs for H_2_/CO_2_ separation [235]. NH_2_-MIL-53 and polymer (VTECTM) MMMs for H_2_/CO_2_ separation have been studied by Musselman et al. as well [236].

The challenge of manufacturing continuous COF membranes has been slightly improved with the introduction of graphene oxide in the preparation process as it will favour the formation of interactions between both materials that gave rise to more stable structures. Ying et al. were pioneers in the preparation of covalent triazine-based framework-1 (CTF-1)/GO ultrathin membranes with H_2_ permeabilities (1.7 × 10^−6^ mol m^−2^s^−1^Pa^−1^) and H_2_/CO_2_ selectivity, exceeding the Robeson’s 2008 upper bound [82]. In a similar approach, Kang and coworkers prepared different composited membranes based on GO and TpPa COFs at different ratios, which were tested for H_2_ purification. The adequate balance between the TpPa-1 COF and the GO allowed reaching a high H_2_/CO_2_ selectivity value of 25.57 and a high H_2_ permeance of 1.067 × 10^−6^ mol m^−2^s^−1^Pa^−1^ [83].

Caro and coworkers reported an innovative COF-COF composite membrane formed by the controlled growth of imine-based COF-LZU-1 and azine-based ACOF-1 layers on a porous surface for its application in gas separation. The formation of interlaced pore channels between the two COF layers, gave rise to better selectivity values for H_2_/CO_2_, H_2_/N_2_, and H_2_/CH_4_ than the COFs separately, exceeding the Robeson upper-bound [169].

In the case of COF MMMs, Wang and coworkers integrated a 2D imine based COF into the poly(vinylamine) (PVAm) polymer matrix, which showed CO_2_/H_2_ selectivity of 15 and CO_2_ permeance of 396 GPU at 0.15 MPa and 10 wt % due to the excellent polymer/COF compatibility and the creation of CO_2_ preferential adsorption sites [124]. In another approach, the high chemical stability of TpBD and TpPa-1 COFs was used by Biswal et al. for the preparation of MMMs that showed high gas permeability for H_2_, CO_2_, and CH_4_ compared to the bare polymers. Particularly, they achieved high hydrogen selectivity from gas mixtures, namely 82.7 for H_2_/N_2_ (with TpBD(40%)@PBI-BuI), up to 165.5 for H_2_/CH_4_ (with TpPa-1(40%)@PBI-BuI), and 3.9 for H_2_/CO_2_ (with TpBD(40%)@PBI-BuI) [141]. Kang et al. demonstrated how the incorporation of the COF NUS-2 into the polybenzimidazole (PBI) polymer matrix gave rise to MMMs with an increase of three orders of magnitude in the H_2_/CO_2_ selectivity with respect to the bare polymer, exceeding the upper-bound reported by Robeson in 2008 [149].

Fu et al. reported on the preparation of two MOF/COF composite membranes, which featured exceptional results in gas separation due to the chemical interactions established between the different components at the interface. Principally, they showed H_2_/CO_2_ selectivity for [COF-300]-[Zn_2_(bdc)_2_(dabco)] and for [COF-300]-[ZIF-8] composites of 12.6 of 13.5, respectively [93].

Kang et al. fabricated a series of COF-GO composite membranes. In addition to the COF’s properties, GO helped to keep the two-dimensional structure of the COF layer. Additionally the rich function groups on the surface of GO interacted with both COF and CO_2_ for the enhancement of H_2_ selectivity. The optimal membrane of TpPa-1-30/GO-10 exhibited a high gas separation permeance of 1.067 × 10^−6^ mol m^−2^s^−1^Pa^−1^ and a H_2_/CO_2_ separation factor of 25.57 [144].

#### 4.1.3. Hydrocarbon Separation

Separation of isomers as well as saturated/unsaturated hydrocarbons with similar physical properties is very important in petroleum refinery, petrochemistry, and natural gas production. Usually these separations are carried out by distillation units, which are extremely costly. So, it is necessary to replace them by energy-efficient and less costly alternative technologies such as the use of membrane separation.

Many MOFs have been reported for hydrocarbon separation [237]. Particularly, the olefin/paraffin separations of ZIF molecular sieve membranes have been reviewed recently by Liu and coworkers [238]. These authors have focused on describing ZIF-based membranes for propylene/propane and ethylene/ethane separations. ZIF-8, whose pore size is between the size of propylene and propane molecules, has been widely studied for this application since the first report in 2012 [239]. Later on, many studies have focused on the effect of the nature of the metal salt as well as membrane fabrication methods used in order to improve the performance of the ZIFs membrane for olefin/paraffin separation [56,240,241,242,243]. For example, the novel membrane synthesis methods all-vapour ligand-induced permselectivation (LIPS) [74] and fast current driven synthesis (FCDS) [244] applied for the preparation of ZIF-8 membranes, showed one of the highest propylene/propane separation factor values (74 and 300, respectively) and propylene permeance (1.6 × 10^−7^ and 1.74 × 10^−8^ mol Pa^−1^m^−2^s^−1^, respectively). Lin et al. showed the beneficial effect on the separation of C_3_H_6_/C_3_H_8_ by preparing MMMs with a filler based on ZIF-8, grown on the surface of carbon nanotubes (CNT). The excellent compatibility between the composite filler and the polymer matrix gave rise to enhance in both C_3_H_6_ permeability and selectivity [245]. Very recently, Nair and coworkers demonstrated that the incorporation of surface-treated nanoparticles of the zeolite MFI during the growth of ZIF-8 MMM originated propylene/propane separation characteristics that exceed the Robeson upper-bound limits. Particularly, the C_3_H_6_ permeability increased dramatically from 371 Barrer in the pure ZIF-8 membrane to 548 Barrer in the ZIF-8/MFI MMM while maintaining the selectivity [246].

**Figure 25:**
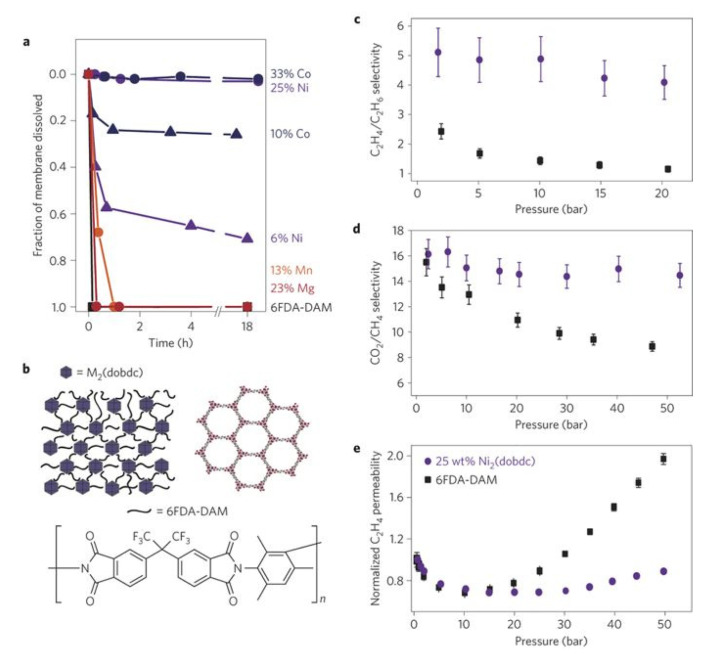
Enhanced membrane stability, reduction in plasticisation, and high mixed-gas selectivity. (**a**), Quantification of membrane stability by Soxhlet extraction in refluxing dichloromethane. The fraction of membrane dissolved corresponds to the mass of membrane remaining after a given period of time in the extractor relative to the initial mass. (**b**) Illustration of the nanocrystal-induced polymer rigidification, along with the structures of M_2_(dobdc) and 6FDA-DAM. Mixed-gas permeation data for a 50:50 C_2_H_4_/C_2_H_6_ (**c**) and a 50:50 CO_2_/CH_4_ mixture (**d**). Error bars correspond to propagation of uncertainty from the mass spectrometer calibration. (**e**), Single-component C_2_H_4_ permeabilities, normalised to the permeability measured at 0.75 bar. Uncertainty in permeability corresponds to propagation of error from uncertainty in the film thickness, area and feed pressure. All permeabilities were collected at 35 °C, and steady-state permeation values were taken after six-time lags. The composition was sampled from permeate that accumulated after steady-state permeation was reached. Permeability and selectivity data correspond to neat 6FDA-DAM (black squares) and 25% Ni_2_(dobdc)/6FDA-DAM (purple circles). Reprinted with permission from [247], Copyright (2016) Springer Nature.

Long and coworkers demonstrated how the nanoparticles of Ni_2_(dobdc) and Co_2_(dobdc) with a size much lower than 100 nm and with high external surface areas were well-dispersed in the 6FDA-DAM polymer matrix due to the interactions between them and the polymer matrix chains. The results showed improvements in ethylene permeability as well as ethylene/ethane selectivity and membrane stability due to the interactions formed and the decrease in the mobility of polymer chains (Figure 25) [247].

In the case of MOF based MMMs, very recently, Liu et al. managed to control both C_3_H_6_/C_3_H_8_ permeability and selectivity in ZIF-8/XLPEO (poly(ethylene oxide), XLPEO) MMM by varying the filler loading as well as the molar ratio of pre-polymers precursors used in the preparation of XLPEO [101].

COF membranes for hydrocarbon recovery have also been studied. Kharul and coworkers were pioneers in the preparation of TpPa-1@SBR (styrene-butadiene rubber, SBR) TFC membranes onto a polyacrylonitrile (PAN) support for propylene/propane separation. The excellent compatibility between the COF and the polymer matrix, allowed obtaining flexible and defect-free TFC membranes with filler loading up to 70 wt %. However, the best performance was reached at 50 wt % with an increase of ca. 8-fold and ca. 12-fold for propylene and propane permeance, respectively. Likewise, slight changes in C_3_H_6_/N_2_ and C_3_H_8_/N_2_ reverse selectivity (20 and 15, respectively) were found for TpPa-1(50)@SBR [248].

MOFs and COFs membranes used for gas separation demand a defect free and a good interface between frameworks and subtract. In some cases, such as CO_2_ removal, the presence of polar groups can enhance the chemical interaction of the membrane with CO_2_ and increase the selectivity. For all gas separations, the chosen best pore size is very important for the best compromise between permeability and selectivity.

### 4.2. Liquid Separation

In this section, different types of separation carried out in liquid phase are addressed. It is highlighted that for this application, membrane stability under the operative conditions is of great importance. As it is well known some types of MOFs and COFs present poor stability in water and acid/basic solutions, which limits their applicability [249]. Recently, Van der Bruggen and coworkers have gathered the main principles for the selection of MOFs to prepare membranes for liquid separation [32]. Here, we will consider MOF and COF membranes employed especially for water treatment, organic solvent nanofiltration, and pervaporation.

#### 4.2.1. Water Treatment

There is a great interest in the development of new technologies for waste water treatment as well as for the desalination of sea water, since the current demand for potable water is increasing [250,251]. Some of the waste that has to be removal from water are salts, metallic ions, dyes, nanoparticles, and organic chemicals. Usually, they are removed by filtration with polymeric membranes, which offer low cost and energy consumption. However, polymer materials do not always meet the necessary requirements of both high permeability and rejection, which limits their use. Kadhom et al. and Lee et al. reviewed MOFs for the membrane desalination and water treatment, showing some remarkable results improving membranes performance [30,252]. Very recently, an example of soluble drug removal using HKUST-1 and ZIF-93 based membranes was reported by Téllez and coworkers. Particularly, these authors studied the elimination of Diclofenac and Naproxen from an aqueous solution having a water permeance in the case of HKUST-1 of 33.1 L m^−2^h^−1^bar^−1^ and 24.9 L m^−2^h^−1^bar^−1^, respectively, with rejections over 98% [253].

**Figure 26:**
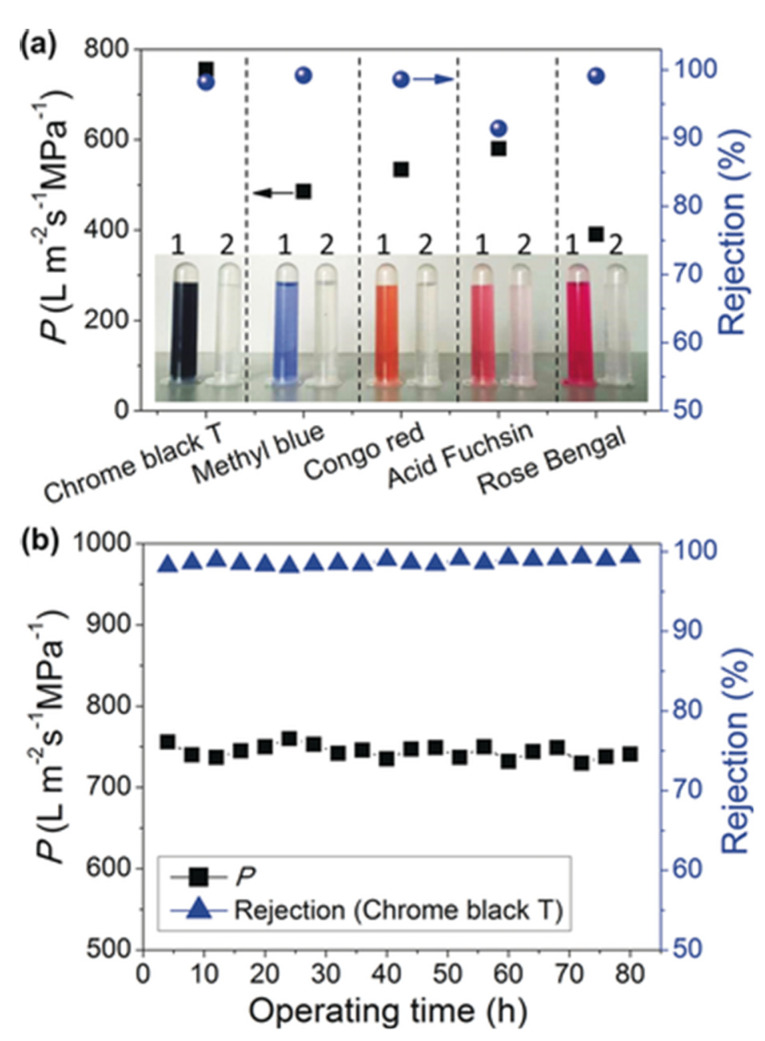
(**a**) Water permeance and rejection rates of the tubular COF-LZU1 membrane in the nanofiltration of different dyes (photographs show the colours of the dye solutions before (1) and after (2) NF). (**b**) Stability test of the tubular COF-LZU1 membrane in a long-time NF of chrome black. Operating pressure: 0.5 MPa; dye concentration: 100 mgL^−1^; room temperature. Reprinted with permission from [78], Copyright (2018) John Wiley and Sons publications.

In the case of COFs, Caro and coworkers reported a stable 2D imine COF-LZU1 membrane grown on alumina tubes by in situ synthesis for dye separation present in water or saline solutions. This excellent 400 nm thick-membrane showed water permeance values (*ca.* 760 L m^−2^h^−1^MPa^−1^) higher than commercial and other reported nanofiltration membranes as well as rejection rates > 90% for dyes larger than 1.2 nm (Figure 26) [78]. Other authors also reported on the preparation of COF based membrane for dye separation showing interesting results [254]. Very recently, Wang and coworkers managed to prepare ultra and nanofiltration membranes based on an imine-linked COF by the modification of the synthesis conditions. These membranes showed high separation efficiencies for dyes from water or organic solution as well as for proteins solutions even higher than other reported membranes prepared from MOFs [255]. In another approach, Xu et al. made use of the excellent hydrolytic and chemical properties of the COFs TpPa-2 for the preparation of a 0.2 wt % TpPa-2@polysulfone (PSf) MMM that showed a significantly high improvement of the performance of the membranes in the removal of organic foulants from water [256]. Similar to MOFs, some COF based membranes have also been tested for water desalination. For example, Wu and coworkers reported a thin-film nanocomposite (TFN) membrane formed by the SNW-1 COF dispersed in polyamide (PA) and supported on a polyether sulfone (PES) substrate, which doubled the flow of water with respect to the pristine membrane and showed a Na_2_SO_4_ rejection above 80% [87].

The permeability during the water treatment mainly depends on the hydrophilicity of the membrane, which could be improved by introducing proper MOFs or COFs structure and also by adding the hydrophilic function on the membrane surface. As for the rejection ability, it is related to the pore size of the membrane as well as the charge of the membrane in the case of salt rejection.

#### 4.2.2. Organic Solvent Nanofiltration

Organic solvent nanofiltration (OSN), also known as solvent resistant nanofiltration (SRNF), is a technology that allows the separation of organic mixtures at the molecular level through the application of a gradient of pressure over a membrane. This technique is used in most processes of food, bio-refinery, petrochemical, and pharmaceutical industries. Interesting reviews have been reported describing the OSN technique by Marchetti et al. and Vandezande et al. [183,257]. An OSN membrane should be robust and stable under operative conditions as well as showing high solvent permeance and high solute rejection. In 2017, Wang and coworkers discussed different polymer based membranes for this application [258]. MOFs and COFs are both chemically stable and structurally well-defined, which could enhance the long-term stability and selectivity for organic solvent nanofiltration membranes.

Livingston et al. prepared thin-film nanocomposite (TFN) membranes with a polyamide (PA) thin-film layer on top of cross-linked polyimide porous supports and a range of 50–150 nm pore size metal-organic framework (MOF) nanoparticles (ZIF-8, MIL-53(Al), NH_2_-MIL-53(Al), and MIL-101(Cr)) via interfacial polymerisation. TFN membrane organic solvent nanofiltration performance was evaluated by solvent permeances (methanol (MeOH) and tetrahydrofuran (THF)) and rejection of styrene oligomers (PS). MeOH and THF permeance increased compared to the same membranes without MOFs, whereas the PS rejection remained higher than 90%. This study showed solvents permeances increased with increasing pore size and porosity of the MOF [259].

Thin film nanocomposite membranes of PA/ZIF-8 (or ZIF-67) @PI has been developed by Coronas et al. via dip-coating of ZIF suspensions on the polyimide supports and then following by interfacial polymerisation of a ultra-thin polyamide layer on the top of the ZIF layer. One of the optimised membrane of PA/ZIF-8@PI with one time ZIF-8 dip-coating in methanol shows 150% improvement of permeance and 90% rejection of sunset yellow (SY) [260].

**Figure 27:**
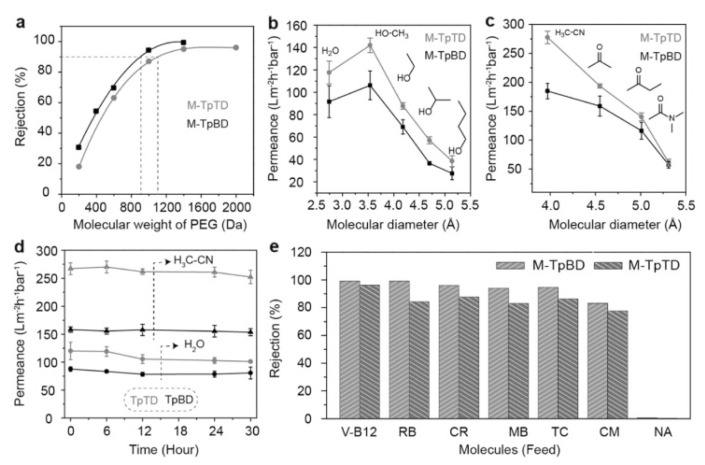
(**a**) Molecular-weight cut-off (MWCO) curve showing that the M-TpBD and M-TpTD had 90% rejection of PEG at 915 Da and 1100 Da, respectively, confirming the pores are in the nanofiltration range; (**b**,**c**) pure solvent permeance versus molecular diameter of different protic (water, methanol, ethanol, 2-propanol, and n-butanol) and aprotic (acetonitrile, acetone, 2-butanone, and N,N′-dimethylacetamide) solvents; (**d**) water and acetonitrile permeance through COMs (M-TpBD and M-TpTD) over time (each 6 h intervals up to 30 h); (**e**) nanofiltration performance of M-TpBD and M-TpTD using different molecules (vitamin B12 (V-B12), rose Bengal (RB), Congo red (CR), curcumin (CM), methylene blue (MB), tetracycline (TC), and nitroaniline (NA)) as a marker in water and acetonitrile (only for CM). A nanofiltration experiment was conducted separately for each aforementioned molecule in a dead-end stirred cell (600 r.p.m.) at ambient conditions and 1 bar upstream pressure. Reprinted with permission from [79], Copyright (2016) John Wiley and Sons publications.

Banerjee and coworkers reported two self-standing and highly stable COF membranes (M-TpBD and M-TpTD) that were tested for the removal of solutes with molecular dimensions larger than 1 nm from aqueous or organic solvents such as dyes, active pharmaceutical ingredients (APIs), and food additives. Moreover, the membranes featured high selectivity towards polar organic solvents, especially in the case of M-TpTD with an acetonitrile flux 2.5 times order magnitude higher than the reference polyamide NF membranes (Figure 27) [79]. In another approach, the same group prepared nanofiltration membranes based on defect-free and self-standing COF thin films. Tp-Bpy, Tp-Azo, Tp-Ttba, and Tp-Tta were chosen to prepare the thin film composite membranes with different pore size via liquid−liquid interfacial polymerisation and then transform to a polyester-3329 nonwoven porous fabric support. The optimised thickness Tp-Bpy membrane exhibited excellent permeance toward both aprotic and protic solvents such as acetonitrile, water, ethanol, and methanol. For solute rejection, Tp-Bpy exhibited high rejection values: 94% for brilliant blue-G (BB), 80% for Congo red (CR), 97% for acid fuchsin (AF), and 98% rhodamine B (RH) [88].

Very recently, Hu and coworkers prepared a new TFN membrane for organic solvent nanofiltration with COFs (SNW-1) nanoparticles in the polyamide skin layer by interfacial polymerisation. The membrane showed improved hydrophilicity and solvent permeance as well as an increase of Rhodamine B rejection (up to 99.4%) compared to COFs-free membranes. The TFN OSN membranes exhibited an excellent N, N-dimethylformamide (DMF) solvent resistance over 100 days. The cross-flow filtration with the TFN membranes using the Rose Bengal/DMF solution at ambient temperature over 7 days, separation performance was maintained. These properties show a strong potential in OSN applications [261].

A novel porous covalent organic triazine-piperazine based TFN membrane (CTP membrane) for OSN was prepared by Peinemann et al. The porous CTP skin layer provided stable porous robust structure, large surface area, well-defined pore topology, and solvent durability. The membrane exhibited excellent separation properties such as selective dye rejection (Reactive black-5, 96.7%) and salt rejection (Na_2_SO_4_, 91.3%). Such a membrane would be a promising candidate for NF applications [262].

**Figure 28:**
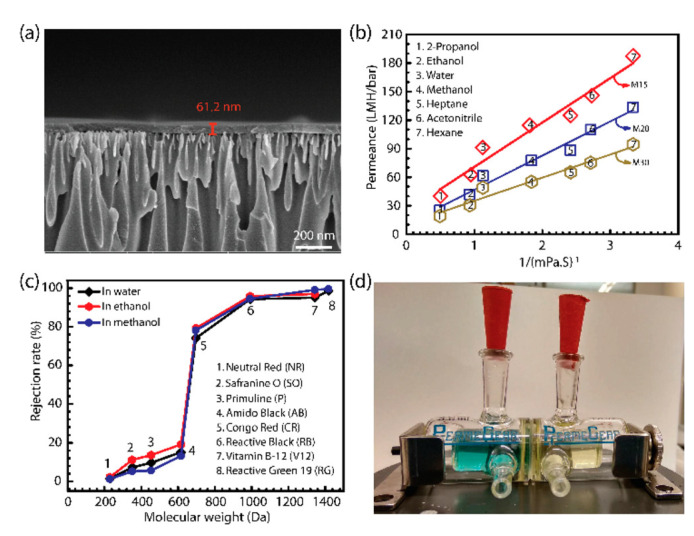
(**a**) Cross-section SEM image of sample M20. (**b**) Permeances of water and a number of polar and nonpolar organic solvents through the three TFP-DHF 2D COF membranes with 15 (M15), 20 (M20), and 30 (M30) layers, plotted with the inverse of their viscosity. (**c**) Rejection rates of various dyes through the M20 membrane vs. their molecular weight. (**d**) Image showing the separation of the mixture dyes of Reactive Green (RG) and Primuline (P). The chamber on the left-hand side contains the mixture of the two dyes, whereas the chamber on the right-hand side is filled with fresh water initially and turned yellow after 1 day of diffusion. Reprinted with permission from [92], Copyright (2018) American Chemical Society.

Shinde et al. prepared a 2D COF membrane by the LB method, which showed permeabilities 5–100 times higher than amorphous membranes and other similar reported materials. The high crystallinity of TFP-DHF 2D COF membrane provided a sharp molecular sieving and long-term rejection of dye molecules in different solvents with an molecular weight cut-off (MWCO) value of approximately 900 Da (Figure 28) [92].

In summary, membrane pore size is the critical factor for separation capacity facing guest molecules. Additionally the stability of MOFs or COFs as well as the substrate in the working solvent is also a necessary requirement for OSN membranes.

#### 4.2.3. Pervaporation

Pervaporation separation is a potential technique for liquid small molecule mixture separation in pharmaceutical and chemical industries. Compare to conventional techniques such as distillation, pervaporation is an energy-saving, and a highly selective technique. MOFs and COFs have good organic compatibility and multi-functionality, which make them a good candidate for the preparation of the pervaporation composite membrane.

Jia et al. have first introduced Zr-MOFs (UiO-66, UiO-66-OH, UiO-66-(OH)_2_, and UiO-67) into polymeric membrane (PVA) for ethanol dehydration via pervaporation. The interactions between Zr-MOFs and PVA matrix and pervaporation performances of MMMs were enhanced successfully by introducing of -OH groups on the organic ligands. For the optimal 1.0 wt.% loading of UiO-66-(OH)2, the water permeance and selectivity increase by 24% and 10% while the swelling degree decreased by 28% in comparison with that of the pristine membrane, making it a potential membrane for ethanol dehydration [263].

Towards industry application, hollow fibre with high packing density could be an ideal substrate for pervaporation mixed-matrix composite membrane. Jin et al. fabricated a novel ceramic hollow fibre supported mixed-matrix composite membrane made of MAF-6 nanoparticles and poly (ether-block-amide) (PEBA) via a facile dip-coating approach. Total flux of 4446 g m^−2^ h and separation factor of 5.6 (feed: 5 wt % ethanol/water, 60 °C) was achieved as optimising result, which showed great advantages over the reported PEBA-based membranes for ethanol/water separation by pervaporation [173].

Recently Steunou et al. prepared ZIF-8 MMM by casting the ZIF-8 collides on the support membrane for isopropanol pervaporation. ZIF-8 collide suspensions were obtained by grafting polyethylene glycol (PEG) at the surface of ZIF-8 nanoparticles (NPs) and then stabilising PEG-g-ZIF-8 NPs by poly(vinyl alcohol) (PVA) to provide a stable casting suspension. Due to the molecular sieving effects of ZIF-8 and the good interfacial properties of the membrane, the pervaporation flux of the MMM was 11 times higher than that of the pure PVA membrane. These MMMs presented a high separation factor up to 7326 [148].

Apart from the alcohol/water pervaporation, other organic liquid mixtures have also been separated by pervaporation using MOF membranes. Caro et al. firstly studied the pervaporation of n-hexane, benzene, and mesitylene as a pure component and binary mixtures on a supported ZIF-8 membrane. The ZIF-8 membranes were prepared by secondary growth on the a-Al2O3 disc membranes. For n-hexane/mesitylene mixture, molecular sieving took place and the n-hexane flux was reduced by pore entrance blocking of increasing concentration of mesitylene [264].

Lin et al. prepared MOF-5 membranes by dip-coating of MOF-5 suspension and secondary growth. Then they studied the pervaporation fluxes and separation factors for toluene, o-xylene, and 1,3,5-triisopropylbenzene (TIPB) pure component and also for 50:50 (mass composition) binary mixtures (toluene/TIPB, o-xylene/TIPB, and toluene/o-xylene) through MOF-5 membranes. Due to the adsorption affinity of guest molecule towards MOF-5 and kinetic diameter of the permeating species, high separation factors for toluene/TIPB, and o xylene/TIPB mixture showed the possibility of separation of these species by pervaporation. However the fouling of the MOF membrane is the main limitation obstacle for their application in organic liquid pervaporation [265].

Jiang et al. used an imine-linked COF TpBD hollow nanospheres (H-TpBD) and sodium alginate (SA) matrices to fabricate water-selective hybrid membranes. The H-TpBD nanospheres provided rich hydrophilic groups and favourable compatibility that rendered the membranes with high water-selective permeability and long-term stability. The optimal performance of the hybrid membranes exhibited a permeation flux of 2170 g m^−2^h^−1^ and separation factor of 2099 when used for dehydrating 90 wt % ethanol aqueous solution at 76 °C [173].

The others similar ethanol/water pervaporation study based on the COF MMM were using different COFs (SNW-1 or COF TpHZ) and also different matrixes such as sodium alginate and poly(ether sulfone) [177,266].

Apart from the dehydration of ethanol via pervaporation, the dehydration of butanol via pervaporation using COFs based MMM were also investigated [267]. Jiang et al. reported a COF membrane through a mixed-dimensional assembly of 2D COF (Schiff-base-type COF TpTGCl) nanosheets and 1D cellulose nanofibers (TEMPO-oxidized CNFs). The COF TpTGCl is chemically stable and can be easily exfoliated to obtain nanosheets. Meanwhile, the intrinsic positive charge of guanidinium units on the TpTGCl framework could help their assembly with negatively charged TEMPO-oxidized CNFs. The membrane enhanced the water adsorption and transport and also provided molecular sieving for improving water solubility and selectivity. The results show a significant improvement of flux (8.53 kg m^−2^h^−1^) with and separation factor (3876) for n-butanol dehydration [161].

Zhang and coworkers reported hydrazone-linked covalent organic-frameworks (COF-42) based membrane for butanol dehydration. The membranes were prepared by dip-coating with a homogeneous suspension of COF-42 and polydimethylsiloxane (PDMS). Since the hydrophilic C=O function is present on the structure of the COF-42, water molecule diffusion was affected and slowed down on the inner wall. The resultant COF-based membrane showed an enhancement of the permeability and selectivity of butanol. The optimal membrane exhibits a high separation factor of 119.7 with a total flux of 3306.7 g m^−2^h^−1^. A “dual membrane process” by coupling the COF-based membrane with a commercial NaA molecular sieving membrane, a 5 wt % feed solution could reach 99.2 wt %, which could be used as the fuel-grade bioalcohol. The high selectivity COF-based membranes could provide a possible non-distillation pathway for biobutanol production [168].

Additionally, COF-LZU1 particles and PEBA have been used for preparing n-butanol dehydration membrane. They showed good permeation flux, excellent running stability, and significant improvement of selectivity [267].

Beside the previous applications, Jiang et al. used COF (SNW-1) based membranes for gasoline pervaporation desulfurisation. The membranes were prepared by spin-coating of casting solutions containing Ag^+^ loaded SNW-1 and Pebax on the Polysulfone (PSf) membranes. The organic nature of SNW-1 optimised the interfacial affinity between filler and polymer, and rendered appropriate free volume properties, molecular sieving ability as well as stability. The membranes displayed enhanced permeation flux, selectivity, and also an anti-swelling property in the separation of the thiophene/n-octane mixture. The optimum performance towards thiophene concentration of 1312 ppm under 60 °C, was achieved with a permeation flux of 16.35 kg m^−2^h^−1^ and enrichment factor of 6.8, which was increased by 78.5% and 30.0% compared with pure Pebax membranes [268].

Membrane pore size is also the main factor for pervaporation application. Some favourable functionalities can also enhance the flux or the selectivity.

### 4.3. Fuel Cells

Polymer electrolyte membranes (PEM) are the key component of fuel cells (FCs), which represent a clean energy alternative. An ideal ion exchange membrane, must meet the following requirements [269]: (a) high ionic conductivity; (b) zero electronic conductivity; (c) low permeability to avoid cross-over; (d) good chemical and thermal stability under fuel cell operating conditions; (e) thin-film processability and stability; (f) high lifetime; and (g) low cost. One of the most common materials used as PEM is Nafion [270,271], developed by DuPont [272], which can reach conductivities values of 10^−1^ S cm^−1^ after its humectation. However, the problems derived from its high cost of production as well as its dehydration at elevated temperatures resulting in a loss of conductivity. To overcome these problems an alternative membrane is required. MOFs and COFs have become ideal candidates for proton conducting applications since they provide an opportunity to characterise the ionic conduction pathway and mechanism, which is difficult to get with other solid electrolytes due to their amorphous nature [193]. Many studies report the design and synthesis of proton-conducting MOFs and COFs, their properties and their operative conditions [273,274,275,276,277,278,279]. The conductivity properties of these materials are usually determined by electrochemical measurements of the processed pellets, which often show poor mechanical flexibility and high thickness limiting their performance under real proton-exchange membrane fuel cell (PEMFC) operating conditions. Therefore, free-standing, flexible membranes based on polycrystalline MOFs and COFs are required. However, so far no example has been reported on the use of MOF membrane directly as a solid electrolyte in a FC. However, Banerjee and coworkers pioneered the use of COFs as solid electrolytes for H_2_/O_2_ fuel cells under real operating conditions. They showed how a bipyridine functionalised COF loaded with H_3_PO_4_ improved both the mechanical and the proton conductivity of the resulting materials. As mentioned before the physical characteristics of the generated COF pellet, limited its performance in the fuel cell [192]. In this regard, Montoro et al. were able to prepare a quasi-transparent and flexible film from an imine-based COF that showed high conductivity values (1.1 × 10^−2^ S cm^−1^ at 323 K). The integration of this novel film-membrane into a single H_2_/O_2_ PEMFC gave rise to a maximum power density peak at 12.95 mW cm^−2^ and a maximum current density of 53.1 mA cm^−2^ [189]. In a subsequent report, Banerjee and coworkers prepared three different free-standing, flexible, porous COF membranes using an amino *p*-toluene sulfonic acid that exhibited superprotonic conductivity values. Particularly, PTSA@TpAzo showed very high proton conducting values (7.8 × 10^−2^ S cm^−1^ at 80 °C under 95% RH) and one of the highest power densities so far reported for crystalline porous organic polymers (24 mW cm^−2^) [80].

**Figure 29:**
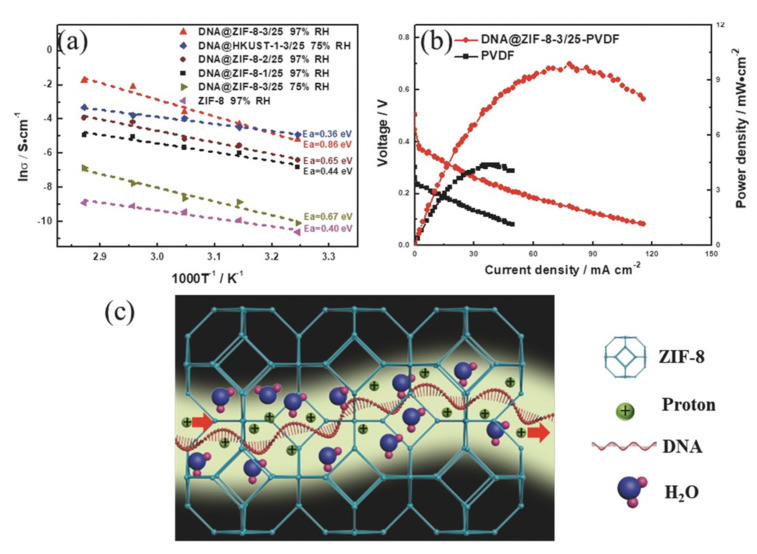
(**a**) Arrhenius plots of the membranes. (**b**) Polarisation curves and power densities of the semi-passive direct methanol fuel cells (DMFCs) using the membranes in 1.0 m methanol at 80 °C. (**c**) The illustration of the proton transportation mechanism through the DNA@ZIF-8 membrane. Reprinted with permission from [281], Copyright (2018) John Wiley and Sons publications.

The use of MMMs as a solid electrolyte in FCs has been highly exploited since they combine the advantages of the polymeric matrix with those from organic/inorganic filler materials [280]. Nafion, sulfonated poly(ether ether ketone) SPEEK, polybenzimidazole (PBI), poly(vinylidene fluoride) (PVDF), and poly(vinylalcohol) (PVA) are some of the most used polymers in the preparation of MMM for this application. The integration of fillers into these polymers is expected to provide better performances due to the synergistic effects of the functional components. Apart from these polymers, Guo et al. fabricated a composite membrane using DNA threaded to ZIF-8 (Figure 29). The low methanol permeability (1.25 × 10^−8^ cm^2^ s^−1^) as well as the high proton conductivity (0.17 S cm^−1^ at 75 °C, under 97% RH) of the DNA@ZIF-8 membrane allowed for the first time, its performance as solid electrolyte in methanol fuel cell leading to a maximum power density of 9.87 mW cm^−2^. The activation energy values obtained (from 0.40 to 0.86 eV) indicated that the proton conductivity follows the Grotthuss mechanism (≤0.4 eV) [281].

In some cases, additives such as graphene oxide (GO), phytic acids, or phosphoric acid are needed to improve the values of ionic conductivity since they can provide additional proton-pathways. For example, Wu et al. prepared a MMM based on Nafion and a zeolitic imidazolate framework–graphene oxide composite (ZIF-8@GO). This hybrid membrane showed better thermal stability as well as an increase of the proton conductivity with the temperature in comparison with recast Nafion. Specifically, at 120 °C and 40% RH the MMM showed conductivity values of 0.28 S cm^−1^ at 120 °C whereas recast Nafion 0.005 S cm^−1^ (Figure 4) due to a synergetic effect of ZIF-8 and GO in promoting proton transfer through a Grotthuss mechanism [282]. In the case of MOFs, an excellent review has been recently published by Escorihuela et al. describing MOF-based MMM used as PEMs for fuel cell applications [283]. As they report, some of the MOFs used for this application are MIL-101 [284,285], MIL-101-SO_3_H [286,287,288], UiO-66-NH_2_ [289,290], UiO-66-SO_3_H [289,290,291], MOF-801 [292], ZIF-8 [282,293,294], ZIF-67 [287], MIL-53-Al [295], and NH_2_-MIL-53 [296]. However, not all of them have been tested under real operating conditions.

Jiang and coworkers prepared a nanohybrid membrane from SPEEK and MIL-101 with phosphotungstic acid encapsulated inside the pore that showed apart from an increase of the thermal stability and enhancement of the proton conductivity with respect to the pristine material. Particularly, for filler loads of 9 wt % the nanohybrid membrane HPW@MIL-101 (9 wt %) exhibited a proton conductivity of 0.272 S cm^−1^ at 65 °C and 100% RH and the pristine SPEEK membrane (0.187 S cm^−1^). Likewise, power density curves obtained after the H_2_/O_2_ single cell performance at 55% RH and 60 °C of SPEEK/HPW@MIL-101 (9 wt %) PEM was in the same order as Nafion 212 (224 mW cm^−2^) and three times higher (235 mW cm^−2^) than that of pristine SPEEK membrane (79 mW cm^−2^) [285]. Zang and coworkers studied the effect of MMM based on chitosan (CS) and MIL-101 its derivatives (S-MIL-101, and acids@MIL-101) in the proton conductivity and interface compatibility. The results showed that the presence of acids inside the MOF pores increase the proton conductivity and better cell performance with CS/H_2_SO_4_@MIL-101-8 with the highest current density (368 mA cm^−2^) and power density (146 mW cm^−2^) similar to the reported CS-based PEM [288]. Tsai et al. prepared two MMM based on Nafion and the 1-D channel microporous MOFs CPO-27(Mg) and MIL-53(Al). The ability of water retention of this MOF leads to better performance than the recast Nafion in terms of proton conductivity and power densities. Specifically, PEM based on CPO-27(Mg) showed a maximum power density of 853 mW cm^−2^ at 50 °C and 568 mW cm^−2^ at 80 °C under 15% RH conditions [295].

In the case of COFs, MMMs prepared from PVDF and the COFs NUS-9 and NUS-10 showed proton conductivities values up to 1.58 × 10^−2^ Scm^−1^ and 5.16 × 10^−3^ Scm^−1^, respectively, due to the presence of pendent sulfonic acid groups into the 1D nanoporous channels of the COFs. The presence of these groups facilitates the adsorption of water molecules as well as serves as favourable pathways for proton conduction following a Grotthuss mechanism [126].

Jiang and coworkers showed the fabrication of a zwitterion-functionalised COF (Z-COF) integrated into Nafion. The presence of both ammonium and sulfonic acid groups in the Z-COF gives rise to an enhancement of water retention property as well as proton conductivity values in the range of 0.22 S cm^−1^ at 80 °C and 100% RH at 10 wt % of Z-COF, which was 57.1% higher than that of recast Nafion membrane. Likewise, Nafion/Z-COF with loads of 45.7 wt % showed the maximum power density of single fuel cell at 80 °C and 50% RH superior also to the recast Nafion membrane [191].

Yin et al. prepared a composite membrane based on Nafion and phosphoric acid-loaded schiff base COF networks with intrinsic amino groups and micropores (H_3_PO_4_@SNW-1). The incorporation of acid groups into the COFs favoured its compatibility with the polymer matrix, optimised hydrophilic domains and generated proton transfer sites. The maximum value of proton conductivity (0.137 S cm^−1^ at 30 °C, 100% RH) and power density was obtained for a load of 15% [190].

In summary, functionalities provided by the MOFs/COFs structures that could increase the interaction with ions (protons) are highly promising to improve the performance of polymer electrolyte membranes. At the same time, well oriented nanopores could serve as nanochannel for ions. Additionally, membrane stability under the working condition of a fuel cell is evidently required.

## 5. Conclusions

MOFs and COFs are an emerging class of crystalline porous materials consisting of orderly arranged pore structures, with high porosity and large surface area. Since various versatile organic linkers could be used in the synthesis of MOFs and COFs, their pore size, shape, and functionalities could be tailored relatively easy. All these aspects make MOFs and COFs ideal candidates for design and synthesis of membranes for separation technologies. Different preparation methods of MOF and COF membranes including free-standing membranes, thin film, MOF/COF composites, and mixed matrix membranes were analysed. Additionally, the main techniques used for characterisation of COF and MOF membranes were summarised. Furthermore, the limitations and advantages of each type of membrane and characterisation methods were also discussed. Finally, the most recent work reported on the use of MOF and COF-based membranes for gas separation (CO_2_ recovery, H_2_ purification and recovery, and hydrocarbon separation), liquid separation (water treatment, organic solvent nanofiltration, and pervaporation), and in fuel cells were reviewed.

## Figures and Tables

**Table 1:** Summary of MOF and COF membrane preparation methods.

	Preparation Method	Thickness	Advantages	Limitations	Applications	References
MOF	In situ or direct growth	300 nm–100 µm	Simple and universal Tunable thickness	Functional substrate surface required Poor heterogeneous nucleation site on the support	Gas separation	[45,46,47,48,49,50,51,52,53]
					Pervaporation	
					OSN	
	Seeded assisted or secondary growth	1–25 µm	Better control of nucleation and crystallinity Various types of supports	Complex procedure Fix small nanosize MOF seeds to the support surface Thicker thickness	Gas separation Water treatment Pervaporation	[54,55,56,57,58,59,60,61,62,63]
	Layer-by-layer assembly	500 nm–2 µm/up to 10 µm	Controllable thickness	Rough surface	Gas separation	[64,65,66,67,68]
			Ultra-thin layer	Small-scale		
	Contra-diffusion or interfacial method	2–25 µm	Various types of supports		Gas separation	[69,70,71]
			Fit for fast reaction		Pervaporation	
			Controllable thickness		OSN	
	Vapour deposition	10–150 nm	Environmentally friendly Time-saving Controllable thickness/Ultra-thin layer	Small-scale	Gas separation	[72,73,74,75]
COF	In situ growth	400 nm–4 µm	Simple Tunable thickness	Functional substrate surface required	Gas storage and separation	[76,77,78]
				(support the initial growth of COFs on the surface)	Water treatment	
	Solution casting	100–700 nm	Simple and scalable	Thicker thickness Less controllable	Water treatment	[79,80]
					OSN	
					Fuel cell (PEM)	
	Layer-by-layer assembly	100–500 nm	Controllable thickness	Extra steps (exfoliation)	Gas separation	[81,82,83]
			Ultra-thin layer			
	Interfacial polymerisation (IP)	2–300 nm / up to 100 µm	Directly forming and scalable Tunable thinkness		Water treatment	[87,88,89,90,91]
					OSN	
	Langmuir−Blodgett (LB) method	3–100 nm	Few COF layers of depostion feasible	Small-scale	Water treatment	[92]
			Can be tranformed to different substrats			
			Turnable thinkness			
